# Crystal structures of 2-[3,5-bis­(bromo­meth­yl)-2,4,6-tri­ethyl­benz­yl]isoindoline-1,3-dione and 2-{5-(bromo­meth­yl)-3-[(1,3-dioxoisoindolin-2-yl)meth­yl]-2,4,6-tri­ethyl­benz­yl}isoindoline-1,3-dione

**DOI:** 10.1107/S205698902100788X

**Published:** 2021-08-10

**Authors:** Manuel Stapf, Betty Leibiger, Anke Schwarzer, Monika Mazik

**Affiliations:** a Institut für Organische Chemie, Technische Universität Bergakademie Freiberg, Leipziger Str. 29, 09599 Freiberg/Sachsen, Germany

**Keywords:** crystal structure, tripodal mol­ecule, phthalimide, halogen bond, hydrogen bonds, hexa­substituted benzene derivative

## Abstract

The title compounds, C_23_H_25_Br_2_NO_2_ (**1**) and C_31_H_29_BrN_2_O_4_ (**2**), crystallize in the space group *P*2_1_/*n* with two (**1-A** and **1-B**) and one mol­ecules, respectively, in the asymmetric unit of the cell. The mol­ecular conformation of these compounds is stabilized by intra­molecular C—H⋯O hydrogen bonds and C—H⋯N or C—H⋯π inter­actions. The crystal structure of **1** features a relatively strong Br⋯O=C halogen bond, which is not observed in the case of **2**. Both crystal structures are characterized by the presence of C—H⋯Br hydrogen bonds and numerous inter­molecular C—H⋯O hydrogen-bonding inter­actions.

## Abstract 2

The title compounds, C_23_H_25_Br_2_NO_2_ (**1**) and C_31_H_29_BrN_2_O_4_ (**2**), crystallize in the space group *P*2_1_/*n* with two and one mol­ecules, respectively, in the asymmetric unit of the cell. The mol­ecular conformation of these compounds is stabilized by intra­molecular C—H⋯O hydrogen bonds and C—H⋯N or C—H⋯π inter­actions. The crystal structure of **1** features a relatively strong Br⋯O=C halogen bond, which is not observed in the case of **2**. Both crystal structures are characterized by the presence of C—H⋯Br hydrogen bonds and numerous inter­molecular C—H⋯O hydrogen-bonding inter­actions.

## Chemical context   

Compounds consisting of a 1,3,5-tris­ubstituted 2,4,6-tri­alkyl­benzene scaffold have been recognized to possess the ability to act as artificial receptors for various neutral and ionic substrates, such as carbohydrates (Mazik, 2009 ▸, 2012 ▸), ion pairs (for example, hydro­nium/hydroxide ions; Stapf *et al.*, 2015 ▸) and ammonium ions (Chin *et al.*, 2002 ▸; Jonah *et al.*, 2017 ▸; Schulze *et al.*, 2018 ▸). In the case of carbohydrate-binding agents (artificial carbohydrate receptors), both acyclic (Kaiser *et al.* 2019 ▸; Stapf *et al.*, 2020*a* ▸, 2020*b* ▸; Köhler *et al.*, 2020 ▸) and macrocyclic compounds (Lippe & Mazik, 2013 ▸, 2015 ▸; Amrhein *et al.*, 2016 ▸; Amrhein & Mazik, 2021 ▸) have been developed. Bromo­methyl- and/or phthalimidomethyl-functionalized tri­alkyl­benzenes are often used as precursors for the syntheses of such compounds . The crystal structures of two representatives of this class of compounds bearing both bromo­methyl- and phthalimidomethyl groups are described in this work.
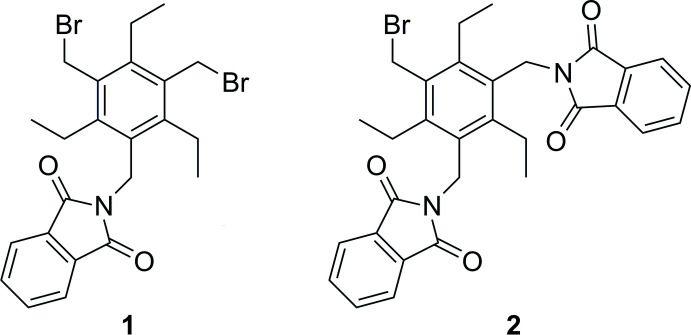


## Structural commentary   

**Figure 1:**
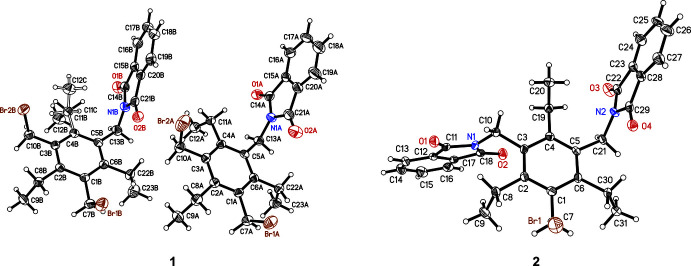
Perspective view of **1** and **2** including the labelling of non-hydrogen atoms. Displacement ellipsoids are drawn at a 50% probability level.

**Figure 2:**
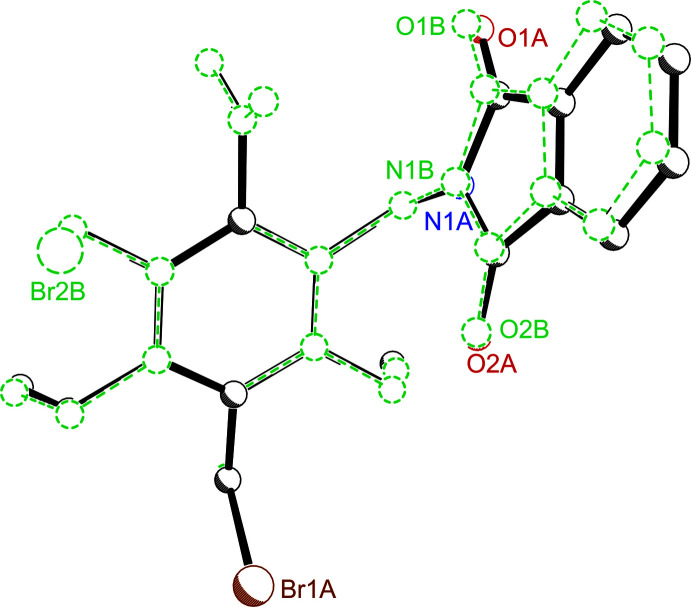
Least-squares overlay of **1-A** and **1-B** with an r.m.s. deviation of 0.0089 Å. The hydrogen atoms are omitted for clarity.

Compounds **1** and **2**, the structures of which are illustrated in Fig. 1 ▸, were found to crystallize in the monoclinic space group *P*2_1_/*n*. In the case of compound **1**, the asymmetric unit of the cell consists of two crystallographically non-equivalent mol­ecules (**1-A** and **1-B**). Mol­ecule **1-A** displays a conformation with a fully alternating arrangement of the substituents above and below the plane of the central benzene ring [*ab′ab′ab′* pattern, *a* = above, *b* = below (*a′*/*b′* = Et above/below); see Koch *et al.*, 2017 ▸; Schulze *et al.*, 2017 ▸]. In mol­ecule **1-B**, one of the ethyl groups is disordered over two positions with an occupancy of 0.820 (6) for the major disorder component; the two disorder positions are related by rotation of approximately 180° about the C4—C11 bond. The mol­ecules display similar conformations, as illustrated by the mol­ecular least-squares overlay shown in Fig. 2 ▸. The dihedral angle between the phthalimide moiety and the benzene ring is 82.27 (14)° (mol­ecule **1-A**) and 83.78 (13)° (mol­ecule **1-B**). The conformation of the mol­ecules appear to be stabilized by intra­molecular C—H⋯O=C hydrogen bonds (Tables 1 ▸ and 2 ▸), which involve ethyl H atoms [*d*(H⋯O) = 2.59, 2.64 Å]. Furthermore, one ethyl group of each mol­ecule participates in the formation of an intra­molecular C—H⋯N bond with H⋯N distances of 2.45 and 2.54 Å, respectively.

The crystal structure of compound **2** contains one mol­ecule in the asymmetric unit of the cell. The two phthalimide groups of the mol­ecule point in opposite directions, showing inclination angles of 70.27 (16) and 79.10 (16)° with respect to the plane of the central aromatic ring. The three-dimensional arrangement of substituents along the periphery of the benzene ring follows an *ab′ba′ab′* pattern, in which the bromo­methyl group, one phthalimidomethyl unit and one ethyl group are directed towards one face of the benzene ring, whereas the three remaining substituents point in the opposite direction. This conformation is stabilized by intra­molecular C_eth­yl_—H⋯O=C (2.45, 2.50 Å) and C_eth­yl_—H⋯π inter­actions [*d*(H⋯*Cg*) 2.80, 2.85 Å].

## Supra­molecular features   

**Figure 3:**
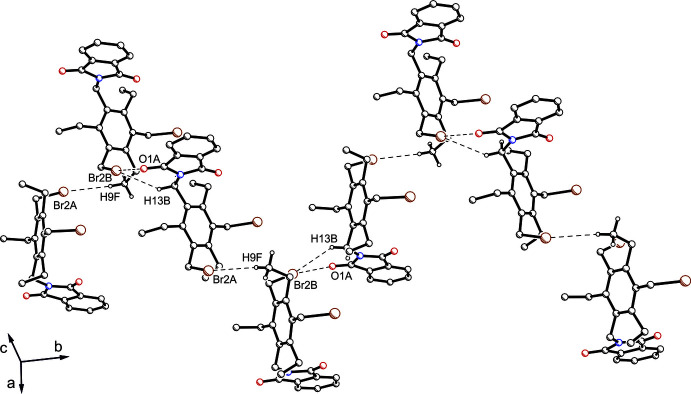
Packing excerpt of **1** showing C—Br⋯O=C and C—H⋯Br halogen and hydrogen bonds, respectively (dashed lines). Hydrogen atoms of subunits that are excluded from inter­molecular inter­actions are omitted for clarity.

In the crystal of compound **1**, the distance of 3.220 (3) Å between Br2*B* and the oxygen atom O1*A* of an adjacent mol­ecules (symmetry code: 1 + *x*, *y*, *z*) is considerably shorter than the sum of the van der Waals radii of the atoms (3.37 Å; Bondi, 1964 ▸); this, as well as the well-defined bond geometry [∠C—Br⋯O = 171.34 (11)°] indicates the presence of a relatively strong Br⋯O halogen bond (Table 3 ▸). This C—Br⋯O=C inter­action is assisted by a C—H⋯Br bond [*d*(H⋯Br) = 2.92 Å, ∠C—H⋯Br = 141.6°], so that atom Br2*B* acts as a bifurcated binding site (see Fig. 3 ▸). The atoms Br1*B* and Br2*A* are involved in the formation of C_eth­yl_—H⋯Br inter­actions with distances of 2.86 and 3.00 Å, respectively (∠C—H⋯Br = 123 and 158°). The two independent mol­ecules are involved in a different way in the mol­ecular association. The phthalimide group of mol­ecule **1-B** participates in the formation of C—H⋯π contacts with H⋯*Cg* distances of 2.62 and 2.96 Å, whereas the phthalimide moiety of the second mol­ecule is involved in the formation of an offset face-to-face inter­action [*d*(*Cg*⋯*Cg*) = 3.75 Å, symmetry code: −*x*, 1 − *y*, 1 − *z*]. In addition, the crystal packing is characterized by the presence of several C—H⋯O hydrogen bonds (2.35–2.43 Å; Table 1 ▸). The different types of non-covalent bonds in the crystal generate a three-dimensional supra­molecular network.

**Figure 4:**
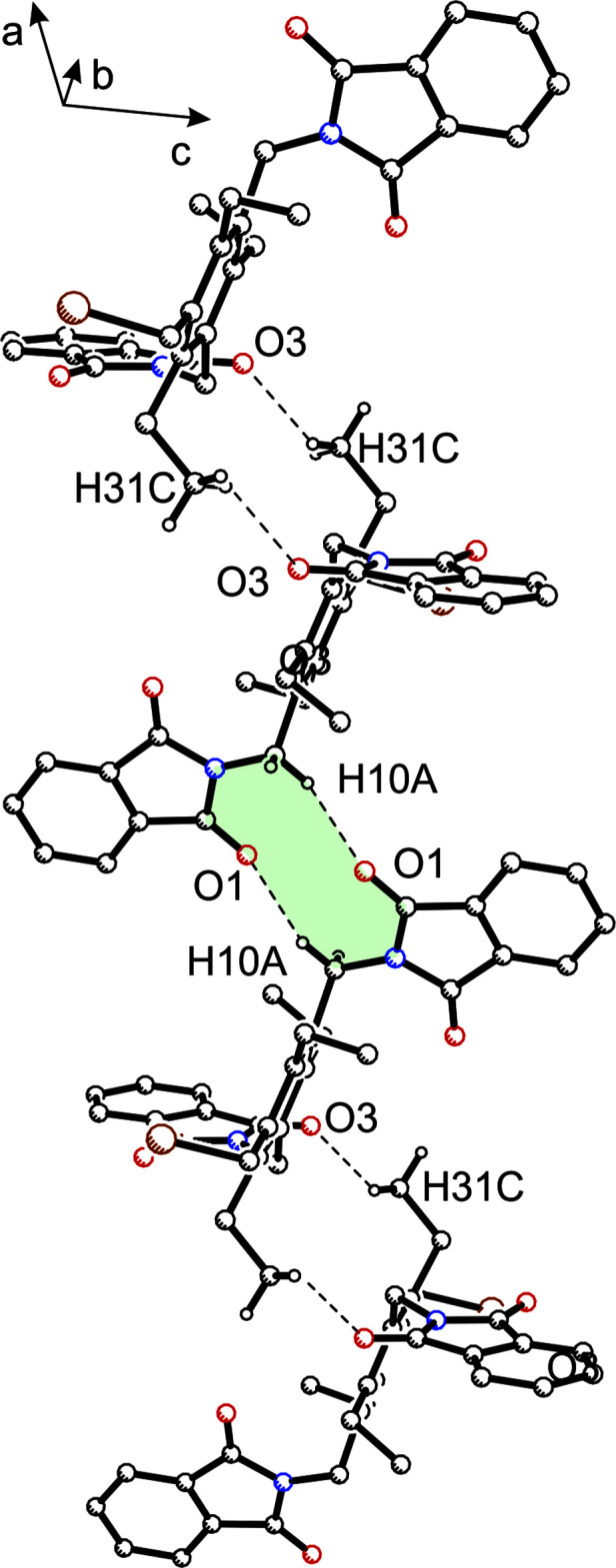
Packing excerpt of **2** showing C—H⋯O hydrogen bonds (dashed lines), which participate in the formation of the supra­molecular ring motif with graph set 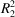
(10). Hydrogen atoms of subunits that are excluded from inter­molecular hydrogen bonding are omitted for clarity.

As a result of the presence of two phthalimide units in compound **2**, its crystal structure is dominated by C—H⋯O bonds [*d*(H⋯O) = 2.49–2.59 Å; Table 2 ▸] in which all oxygen atoms participate. The fragment of the packing structure shown in Fig. 4 ▸ shows that atoms O1 and H10*A* take part in the formation of an inversion-symmetric supra­molecular ring motif with graph-set motif 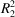
(10) (Etter, 1990 ▸; Bernstein *et al.*, 1995 ▸; such a ten-membered supra­molecular motif has, for example, been recognized in some crystal structures of fluorene derivatives bearing phthalimidomethyl groups, see Seidel *et al.*, 2021 ▸). In addition, the mol­ecules are linked by two C—H⋯π inter­actions [*d*(H⋯*Cg* = 2.84, 2.88 Å] with the C1–C6 and C12–C17 rings acting as acceptors.

## Database survey   

A search in the Cambridge Structural Database (CSD, Version 5.41, update of November 2019; Groom *et al.*, 2016 ▸) for 2-benzyl­isoindoline-1,3-dione resulted in 48 hits. Regarding the description of crystal structures of tri- to hexa­substituted benzene derivatives, the number of hits could be reduced to three relevant entries. This includes two hexa­substituted benzene derivatives consisting of three isoindoline-1,3-dione groups (phthalimidomethyl groups) and either meth­oxy (IDOBIO; Rosien *et al.*, 2013 ▸) or bromo­methyl groups (LOFBIT; Koch *et al.*, 2014 ▸) in each of the 2-, 4- and 6-positions of the benzene ring. Furthermore, a 1,3,5-tris­ubstituted benzene derivative, namely 3,5-bis­(phthalimidometh­yl)phenyl-*tert*-butyl­dimethyl­silyl ether (WIKRAK; Domínguez *et al.*, 2007 ▸), has been found. In the case of IDOBIO and LOFBIT, the mol­ecules adopt a conformation in which two phthalimidomethyl groups and one meth­oxy or bromo­methyl group are directed towards one face of the benzene ring. The phthalimidomethyl groups of the 1,3,5-tris­ubstituted benzene derivative adopt a *trans* geometry.

## Synthesis and crystallization   

A suspension of 1,3,5-tris­(bromo­meth­yl)-2,4,6-tri­ethyl­benzene (1.00 g, 2.27 mmol) and potassium phthalimide (0.84 g, 4.54 mmol) in a solvent mixture *N*,*N*-di­methyl­formamide/1,4-dioxane (15 ml, 2:1, *v*/*v*) was stirred at ambient temperature for 24 h. Afterwards, the reaction mixture was poured into 50 ml of water. The white precipitate was filtered off, washed several times with water and finally suspended in water. After extraction with chloro­form (five times) and evaporation of the organic solvent, the crude product was purified by column chromatography (SiO_2_; toluene/ethyl acetate). Compounds **1** and **2** were obtained as white solids.

Compound **1**: Yield: 27%; m.p. 482 K (decomposition; toluene/ethyl acetate); *R_f_* = 0.68 (SiO_2_; toluene/ethyl acetate 10:1 *v*/*v*); ^1^H NMR (500 MHz, CDCl_3_): 1.16 (*t*, 6H, *J* = 7.6 Hz), 1.35 (*t*, 3H, *J* = 7.6 Hz), 2.94 (*q*, 2H, *J* = 7.6 Hz), 3.03 (*q*, 4H, *J* = 7.6 Hz), 4.61 (*s*, 4H), 4.92 (*s*, 2H), 7.69–7.71 (*m*, 2H), 7.72–7.83 (*m*, 2H) ppm; ^13^C NMR (500 MHz, CDCl_3_): 15.6, 15.7, 22.8, 23.0, 29.1, 37.0, 123.3, 130.6, 131.9, 132.1, 134.1, 144.2, 145.8, 168.1 ppm; IR (ATR): 2969, 1709, 1491, 1454, 1392, 592 cm^−1^; LC–MS (ESI): calculated for C_23_H_25_Br_2_NO_2_Na (*M* + Na)^+^: 530.01, found: 530.21.

Compound **2**: Yield: 40%; m.p. 494–495 K (toluene/ethyl acetate); *R_f_* = 0.48 (SiO_2_; toluene/ethyl acetate 10:1 *v*/*v*); ^1^H NMR (500 MHz, CDCl_3_): 0.97 (*t*, 3H, *J* = 7.6 Hz), 1.14 (*t*, 6H, *J* = 7.6 Hz), 3.00 (*q*, 4H, *J* = 7.6 Hz), 3.18 (*q*, 2H, *J* = 7.6 Hz), 4.63 (*s*, 2H), 4.94 (*s*, 4H), 7.68–7.70 (*m*, 4H), 7.71–7.83 (*m*, 4H) ppm; ^13^C NMR (500 MHz, CDCl_3_): 15.7, 15.8, 23.0, 23.5, 29.7, 37.3, 123.3, 130.0, 131.7, 131.9, 134.0, 144.8, 146.5, 168.2 ppm; IR (ATR): 2962, 1700, 1498, 1463, 1392, 528 cm^−1^; LC–MS (ESI): calculated for C_31_H_30_BrN_2_O_4_ (*M* + H)^+^: 575.14, found: 575.06.

Single crystals suitable for X-ray diffraction were obtained by crystallization of the respective compound from toluene/ethyl acetate (**1**) and toluene (**2**).

## Refinement   

Crystal data, data collection and structure refinement details are summarized in Table 4 ▸. All H atoms were positioned geometrically and refined as riding, with C—H = 0.95–0.99 Å, and with *U*_iso_(H) = 1.5 *U*_eq_(C) for methyl groups or *U*_iso_(H) = 1.2 *U*_eq_(C) otherwise. For compound **1**, one ethyl group (C11*B*–C12*B*/C11*C*–C12*C*) in **1-B** was refined in two positions using EADP and EXYZ restraints.

## Supplementary Material

Crystal structure: contains datablock(s) 1, 2, global. DOI: 10.1107/S205698902100788X/zq2264sup1.cif

Structure factors: contains datablock(s) 1. DOI: 10.1107/S205698902100788X/zq22641sup2.hkl

Structure factors: contains datablock(s) 2. DOI: 10.1107/S205698902100788X/zq22642sup3.hkl

Supporting information file. DOI: 10.1107/S205698902100788X/zq22641sup4.cml

Supporting information file. DOI: 10.1107/S205698902100788X/zq22642sup5.cml

CCDC references: 2100927, 2100926

Additional supporting information:  crystallographic information; 3D view; checkCIF report

## Figures and Tables

**Table 1:** Hydrogen-bond geometry (Å, °) for **1** [Chem scheme1] *Cg*4 is the centroid of the C15*B*–C20*B* ring.

*D*—H⋯*A*	*D*—H	H⋯*A*	*D*⋯*A*	*D*—H⋯*A*
C10*A*—H10*B*⋯O2*B*	0.99	2.35	3.223 (4)	147
C11*A*—H11*A*⋯N1*A*	0.99	2.54	3.283 (4)	132
C13*A*—H13*B*⋯Br2*B* ^i^	0.99	2.92	3.746 (3)	142
C13*A*—H13*B*⋯O1*A*	0.99	2.52	2.914 (4)	103
C9*B*—H9*F*⋯Br2*A* ^ii^	0.98	3.00	3.921 (4)	158
C11*B*—H11*D*⋯N1*B*	0.99	2.45	3.207 (4)	133
C12*B*—H12*D*⋯Br1*B* ^ii^	0.98	2.86	3.499 (4)	123
C13*B*—H13*D*⋯O1*B*	0.99	2.53	2.928 (4)	104
C22*B*—H22*D*⋯O2*B*	0.99	2.64	3.322 (4)	126
C23*B*—H23*E*⋯O2*A* ^iii^	0.98	2.43	3.226 (5)	138
C22*A*—H22*B*⋯O2*A*	0.99	2.59	3.278 (4)	126
C9*B*—H9*D*⋯*Cg*4^iv^	0.98	2.96	3.731 (5)	137
C23*B*—H23*D*⋯*Cg*4^v^	0.98	2.92	3.542 (5)	122
C12*C*—H12*I*⋯N1*B*	0.98	2.56	3.24 (2)	126

Symmetry codes: (i) x-1, y, z; (ii) -x+{\script{3\over 2}}, y-{\script{1\over 2}}, -z+{\script{1\over 2}}; (iii) -x+{\script{1\over 2}}, y-{\script{1\over 2}}, -z+{\script{1\over 2}}; (iv) x+{\script{1\over 2}}, -y+{\script{1\over 2}}, z-{\script{1\over 2}}; (v) x-{\script{1\over 2}}, -y+{\script{1\over 2}}, z-{\script{1\over 2}}.

**Table 2:** Hydrogen-bond geometry (Å, °) for **2** [Chem scheme1] *Cg*1 and *Cg*3 are the centroids of the C1–C6 and C12–C17 rings, respectively.

*D*—H⋯*A*	*D*—H	H⋯*A*	*D*⋯*A*	*D*—H⋯*A*
C10—H10*A*⋯O1	0.99	2.49	2.896 (5)	104
C10—H10*A*⋯O1^i^	0.99	2.49	3.173 (5)	126
C19—H19*B*⋯O3	0.99	2.45	3.373 (5)	154
C21—H21*B*⋯O3	0.99	2.47	2.897 (5)	105
C25—H25⋯O4^ii^	0.95	2.58	3.237 (5)	127
C30—H30*B*⋯O4	0.99	2.50	3.346 (5)	144
C31—H31*B*⋯O2^iii^	0.98	2.59	3.298 (5)	129
C31—H31*C*⋯O3^iv^	0.98	2.53	3.334 (5)	139
C26—H26⋯*Cg*1^ii^	0.95	2.84	3.529 (5)	130
C31—H31*A*⋯*Cg*3^v^	0.98	2.88	3.394 (5)	113

Symmetry codes: (i) -x, -y, -z+1; (ii) -x+{\script{1\over 2}}, y+{\script{1\over 2}}, -z+{\script{3\over 2}}; (iii) x+{\script{1\over 2}}, -y+{\script{1\over 2}}, z+{\script{1\over 2}}; (iv) -x+1, -y+1, -z+1; (v) -x+1, -y, -z+1.

**Table 3:** Halogen bonds in **1**

C—*X*⋯*Y*—C	symmetry code	C—*X*/*Y*	*X*⋯*Y*	C—*X*/*Y*⋯*Y*/*X*
C10*B*—Br2*B*⋯O1*A*—C14*A*	1 + *x*, *y*, *z*	1.980 (3)/1.210 (4)	3.220 (3)	129.0 (2)/171.35 (11)

**Table 4:** Experimental details

	**1**	**2**
Crystal data		
Chemical formula	C_23_H_25_Br_2_NO_2_	C_31_H_29_BrN_2_O_4_
*M*_r_	507.26	573.47
Crystal system, space group	Monoclinic, *P*2_1_/*n*	Monoclinic, *P*2_1_/*n*
Temperature (K)	153	153
*a*, *b*, *c* (Å)	13.367 (2), 19.966 (3), 16.919 (4)	12.899 (2), 12.9748 (15), 16.763 (3)
β (°)	106.099 (15)	109.168 (13)
*V* (Å^3^)	4338.5 (14)	2649.9 (7)
*Z*	8	4
Radiation type	Mo *K*α	Mo *K*α
μ (mm^−1^)	3.76	1.59
Crystal size (mm)	0.40 × 0.23 × 0.17	0.18 × 0.18 × 0.15

Data collection		
Diffractometer	Stoe IPDS 2T	Stoe IPDS 2
Absorption correction	Integration	Integration
*T*_min_, *T*_max_	0.324, 0.472	0.695, 0.844
No. of measured, independent and observed [*I* > 2σ(*I*)] reflections	48044, 8523, 5961	26391, 4941, 3442
*R*_int_	0.067	0.115
(sin θ/λ)_max_ (Å^−1^)	0.617	0.606

Refinement		
*R*[*F*^2^ > 2σ(*F* ^2^)], *wR*(*F* ^2^), *S*	0.036, 0.080, 1.02	0.056, 0.129, 1.12
No. of reflections	8523	4941
No. of parameters	516	346
No. of restraints	5	0
H-atom treatment	H-atom parameters constrained	H-atom parameters constrained
Δρ_max_, Δρ_min_ (e Å^−3^)	0.78, −0.85	0.38, −0.67

Computer programs: *X-AREA* and *X-RED* (Stoe, 2009 ▸), *SHELXT2018/2* (Sheldrick, 2015 ▸a), *SHELXL2018/3* (Sheldrick, 2015 ▸b), *XP* (Sheldrick, 2008 ▸), *WinGX* (Farrugia, 2012 ▸), *publCIF* (Westrip, 2010 ▸) and *shelXle* (Hübschle *et al.*, 2011 ▸).

## supplementary crystallographic information

### 2-[3,5-Bis(bromomethyl)-2,4,6-triethylbenzyl]isoindoline-1,3-dione (1) Crystal data

**Table:**

C_23_H_25_Br_2_NO_2_	*F*(000) = 2048
*M**_r_* = 507.26	*D*_x_ = 1.553 Mg m^−^^3^
Monoclinic, *P*2_1_/*n*	Mo *K*α radiation, λ = 0.71073 Å
*a* = 13.367 (2) Å	Cell parameters from 2236 reflections
*b* = 19.966 (3) Å	θ = 2.6–25.7°
*c* = 16.919 (4) Å	µ = 3.76 mm^−^^1^
β = 106.099 (15)°	*T* = 153 K
*V* = 4338.5 (14) Å^3^	Piece, colorless
*Z* = 8	0.40 × 0.23 × 0.17 mm

### 2-[3,5-Bis(bromomethyl)-2,4,6-triethylbenzyl]isoindoline-1,3-dione (1) Data collection

**Table:**

STOE IPDS 2T diffractometer	8523 independent reflections
Radiation source: sealed X-ray tube, 12 x 0.4 mm long-fine focus	5961 reflections with *I* > 2σ(*I*)
Plane graphite monochromator	*R*_int_ = 0.067
Detector resolution: 6.67 pixels mm^-1^	θ_max_ = 26.0°, θ_min_ = 2.6°
rotation method scans	*h* = −16→16
Absorption correction: integration	*k* = −24→24
*T*_min_ = 0.324, *T*_max_ = 0.472	*l* = −20→20
48044 measured reflections	

### 2-[3,5-Bis(bromomethyl)-2,4,6-triethylbenzyl]isoindoline-1,3-dione (1) Refinement

**Table:**

Refinement on *F*^2^	5 restraints
Least-squares matrix: full	Hydrogen site location: inferred from neighbouring sites
*R*[*F*^2^ > 2σ(*F*^2^)] = 0.036	H-atom parameters constrained
*wR*(*F*^2^) = 0.080	*w* = 1/[σ^2^(*F*_o_^2^) + (0.0287*P*)^2^ + 3.7348*P*] where *P* = (*F*_o_^2^ + 2*F*_c_^2^)/3
*S* = 1.02	(Δ/σ)_max_ = 0.001
8523 reflections	Δρ_max_ = 0.78 e Å^−^^3^
516 parameters	Δρ_min_ = −0.85 e Å^−^^3^

### 2-[3,5-Bis(bromomethyl)-2,4,6-triethylbenzyl]isoindoline-1,3-dione (1) Special details

**Table:**

Geometry. All esds (except the esd in the dihedral angle between two l.s. planes) are estimated using the full covariance matrix. The cell esds are taken into account individually in the estimation of esds in distances, angles and torsion angles; correlations between esds in cell parameters are only used when they are defined by crystal symmetry. An approximate (isotropic) treatment of cell esds is used for estimating esds involving l.s. planes.

### 2-[3,5-Bis(bromomethyl)-2,4,6-triethylbenzyl]isoindoline-1,3-dione (1) Fractional atomic coordinates and isotropic or equivalent isotropic displacement parameters (Å^2^)

**Table:**

	*x*	*y*	*z*	*U*_iso_*/*U*_eq_	Occ. (<1)
Br1A	0.16416 (3)	0.64628 (2)	0.09609 (2)	0.04094 (10)	
Br2A	0.55611 (3)	0.52669 (2)	0.36556 (3)	0.05486 (13)	
O1A	0.1498 (2)	0.36605 (13)	0.49979 (14)	0.0395 (6)	
O2A	0.1109 (2)	0.57148 (12)	0.37508 (16)	0.0469 (7)	
N1A	0.1343 (2)	0.46167 (13)	0.41932 (16)	0.0279 (6)	
C1A	0.2142 (2)	0.51420 (15)	0.16832 (19)	0.0235 (7)	
C2A	0.3218 (2)	0.50277 (15)	0.19315 (19)	0.0246 (7)	
C3A	0.3659 (2)	0.46791 (15)	0.26682 (19)	0.0241 (7)	
C4A	0.3024 (2)	0.44455 (15)	0.31516 (18)	0.0229 (7)	
C5A	0.1951 (2)	0.45732 (15)	0.29062 (18)	0.0225 (7)	
C6A	0.1501 (2)	0.49312 (15)	0.21738 (18)	0.0230 (7)	
C7A	0.1661 (3)	0.54733 (15)	0.0867 (2)	0.0287 (7)	
H7A	0.093934	0.530854	0.064312	0.034*	
H7B	0.205959	0.534699	0.047632	0.034*	
C8A	0.3894 (3)	0.52472 (18)	0.1391 (2)	0.0340 (8)	
H8A	0.459884	0.535976	0.174052	0.041*	
H8B	0.359230	0.565484	0.108240	0.041*	
C9A	0.3974 (3)	0.4693 (2)	0.0781 (2)	0.0448 (10)	
H9A	0.441493	0.484750	0.044129	0.067*	
H9B	0.327785	0.458744	0.042768	0.067*	
H9C	0.428167	0.429159	0.108595	0.067*	
C10A	0.4810 (3)	0.45441 (16)	0.2934 (2)	0.0308 (8)	
H10A	0.507217	0.450900	0.244241	0.037*	
H10B	0.494319	0.411194	0.323135	0.037*	
C11A	0.3497 (3)	0.40134 (16)	0.39013 (19)	0.0269 (7)	
H11A	0.308225	0.406023	0.430053	0.032*	
H11B	0.421453	0.416754	0.417113	0.032*	
C12A	0.3520 (3)	0.32768 (17)	0.3655 (2)	0.0347 (8)	
H12A	0.392091	0.301699	0.412842	0.052*	
H12B	0.384727	0.323864	0.320503	0.052*	
H12C	0.280706	0.310351	0.347287	0.052*	
C13A	0.1249 (3)	0.42863 (16)	0.33988 (19)	0.0270 (7)	
H13A	0.051651	0.431931	0.306061	0.032*	
H13B	0.141356	0.380507	0.350038	0.032*	
C14A	0.1440 (3)	0.42633 (18)	0.4930 (2)	0.0301 (8)	
C15A	0.1469 (3)	0.47882 (19)	0.5564 (2)	0.0329 (8)	
C16A	0.1600 (3)	0.4726 (2)	0.6405 (2)	0.0425 (9)	
H16A	0.165391	0.430161	0.666754	0.051*	
C17A	0.1650 (3)	0.5332 (2)	0.6847 (2)	0.0499 (11)	
H17A	0.174875	0.531280	0.742489	0.060*	
C18A	0.1561 (3)	0.5948 (2)	0.6471 (3)	0.0508 (10)	
H18A	0.160339	0.634344	0.679134	0.061*	
C19A	0.1409 (3)	0.5999 (2)	0.5630 (2)	0.0464 (10)	
H19A	0.133774	0.642245	0.536402	0.056*	
C20A	0.1366 (3)	0.54083 (18)	0.5190 (2)	0.0345 (8)	
C21A	0.1250 (3)	0.53045 (18)	0.4296 (2)	0.0328 (8)	
C22A	0.0342 (2)	0.50790 (17)	0.1894 (2)	0.0286 (7)	
H22A	0.022597	0.549713	0.156644	0.034*	
H22B	0.008679	0.515234	0.238308	0.034*	
C23A	−0.0282 (3)	0.45160 (19)	0.1379 (2)	0.0377 (9)	
H23A	−0.101033	0.465459	0.116564	0.057*	
H23B	−0.024207	0.411552	0.172112	0.057*	
H23C	0.000531	0.441538	0.091847	0.057*	
Br1B	0.67396 (3)	0.39932 (2)	0.09585 (2)	0.04119 (10)	
Br2B	1.04422 (3)	0.25251 (2)	0.36780 (2)	0.03582 (9)	
O1B	0.6272 (2)	0.11607 (13)	0.48916 (16)	0.0435 (7)	
O2B	0.61521 (19)	0.32716 (12)	0.38071 (14)	0.0350 (6)	
N1B	0.6197 (2)	0.21507 (14)	0.41532 (16)	0.0300 (6)	
C1B	0.7055 (2)	0.26581 (14)	0.16601 (18)	0.0225 (7)	
C2B	0.8113 (2)	0.24748 (15)	0.19156 (18)	0.0234 (6)	
C3B	0.8472 (2)	0.20833 (15)	0.26292 (18)	0.0236 (7)	
C4B	0.7787 (3)	0.18849 (15)	0.30865 (19)	0.0252 (7)	
C5B	0.6737 (2)	0.20943 (15)	0.28466 (19)	0.0245 (7)	
C6B	0.6371 (2)	0.24923 (16)	0.21354 (18)	0.0249 (7)	
C7B	0.6642 (3)	0.30068 (16)	0.0850 (2)	0.0295 (7)	
H7C	0.590476	0.287854	0.060999	0.035*	
H7D	0.703943	0.285679	0.046832	0.035*	
C8B	0.8845 (3)	0.26902 (16)	0.14208 (19)	0.0279 (7)	
H8C	0.954519	0.277115	0.179979	0.033*	
H8D	0.859366	0.311679	0.113665	0.033*	
C9B	0.8929 (3)	0.21651 (19)	0.0782 (2)	0.0359 (8)	
H9D	0.939746	0.232899	0.047079	0.054*	
H9E	0.823776	0.208315	0.040526	0.054*	
H9F	0.920501	0.174702	0.106241	0.054*	
C10B	0.9591 (3)	0.18730 (17)	0.2891 (2)	0.0300 (7)	
H10C	0.985816	0.183927	0.240246	0.036*	
H10D	0.964949	0.142577	0.315289	0.036*	
C11B	0.8180 (3)	0.14231 (16)	0.3819 (2)	0.0311 (8)	0.820 (6)
H11C	0.891146	0.153595	0.410628	0.037*	0.820 (6)
H11D	0.775821	0.148948	0.421073	0.037*	0.820 (6)
C12B	0.8113 (4)	0.0699 (2)	0.3550 (3)	0.0406 (12)	0.820 (6)
H12D	0.848945	0.041825	0.401198	0.061*	0.820 (6)
H12E	0.842563	0.064919	0.309354	0.061*	0.820 (6)
H12F	0.738156	0.056104	0.337049	0.061*	0.820 (6)
C11C	0.8180 (3)	0.14231 (16)	0.3819 (2)	0.0311 (8)	0.180 (6)
H11E	0.758897	0.115163	0.388690	0.037*	0.180 (6)
H11F	0.869688	0.111156	0.370233	0.037*	0.180 (6)
C12C	0.8641 (17)	0.1758 (10)	0.4567 (12)	0.0406 (12)	0.180 (6)
H12G	0.932253	0.193439	0.455771	0.061*	0.180 (6)
H12H	0.872996	0.144431	0.502612	0.061*	0.180 (6)
H12I	0.819208	0.212918	0.463481	0.061*	0.180 (6)
C13B	0.5994 (3)	0.18597 (17)	0.3324 (2)	0.0330 (8)	
H13C	0.527408	0.197432	0.300662	0.040*	
H13D	0.603806	0.136584	0.337432	0.040*	
C14B	0.6311 (3)	0.17670 (19)	0.4873 (2)	0.0333 (8)	
C15B	0.6500 (3)	0.22625 (19)	0.5562 (2)	0.0335 (8)	
C16B	0.6689 (3)	0.2166 (2)	0.6403 (2)	0.0412 (9)	
H16B	0.671296	0.173089	0.663423	0.049*	
C17B	0.6840 (3)	0.2739 (2)	0.6891 (2)	0.0480 (10)	
H17B	0.696999	0.269143	0.746899	0.058*	
C18B	0.6808 (3)	0.3374 (2)	0.6561 (2)	0.0464 (10)	
H18B	0.691568	0.375124	0.691637	0.056*	
C19B	0.6621 (3)	0.3470 (2)	0.5715 (2)	0.0398 (9)	
H19B	0.659735	0.390576	0.548338	0.048*	
C20B	0.6471 (2)	0.29004 (18)	0.5229 (2)	0.0305 (8)	
C21B	0.6258 (3)	0.28343 (18)	0.4321 (2)	0.0300 (7)	
C22B	0.5264 (2)	0.27532 (17)	0.1873 (2)	0.0303 (7)	
H22C	0.525540	0.319108	0.159625	0.036*	
H22D	0.502198	0.282707	0.236834	0.036*	
C23B	0.4509 (3)	0.2279 (2)	0.1292 (3)	0.0509 (10)	
H23D	0.382770	0.249568	0.109135	0.076*	
H23E	0.443652	0.186717	0.158665	0.076*	
H23F	0.477779	0.216890	0.082429	0.076*	

### 2-[3,5-Bis(bromomethyl)-2,4,6-triethylbenzyl]isoindoline-1,3-dione (1) Atomic displacement parameters (Å^2^)

**Table:**

	*U*^11^	*U*^22^	*U*^33^	*U*^12^	*U*^13^	*U*^23^
Br1A	0.0464 (2)	0.02807 (18)	0.0440 (2)	0.00305 (16)	0.00543 (18)	0.01000 (16)
Br2A	0.0321 (2)	0.0477 (2)	0.0728 (3)	−0.00696 (19)	−0.0055 (2)	−0.0089 (2)
O1A	0.0469 (16)	0.0415 (16)	0.0306 (13)	0.0038 (12)	0.0115 (12)	0.0086 (11)
O2A	0.071 (2)	0.0318 (14)	0.0397 (15)	−0.0018 (13)	0.0188 (14)	−0.0002 (12)
N1A	0.0324 (16)	0.0302 (15)	0.0227 (15)	0.0005 (12)	0.0101 (12)	−0.0010 (11)
C1A	0.0253 (17)	0.0222 (16)	0.0208 (16)	0.0018 (13)	0.0025 (14)	−0.0012 (12)
C2A	0.0266 (17)	0.0223 (16)	0.0258 (17)	−0.0007 (13)	0.0088 (14)	0.0017 (13)
C3A	0.0245 (17)	0.0196 (15)	0.0269 (17)	0.0006 (13)	0.0050 (14)	−0.0006 (13)
C4A	0.0255 (17)	0.0202 (15)	0.0212 (16)	0.0011 (13)	0.0038 (14)	0.0005 (12)
C5A	0.0266 (17)	0.0221 (16)	0.0197 (16)	−0.0018 (13)	0.0077 (14)	−0.0040 (12)
C6A	0.0236 (17)	0.0225 (16)	0.0228 (16)	−0.0021 (13)	0.0064 (13)	−0.0041 (12)
C7A	0.0315 (19)	0.0252 (17)	0.0277 (18)	−0.0023 (14)	0.0053 (15)	0.0015 (13)
C8A	0.0294 (19)	0.040 (2)	0.036 (2)	0.0003 (16)	0.0132 (16)	0.0146 (16)
C9A	0.045 (2)	0.060 (3)	0.037 (2)	0.012 (2)	0.0224 (19)	0.0108 (19)
C10A	0.0281 (18)	0.0292 (18)	0.0348 (19)	0.0012 (14)	0.0085 (16)	0.0042 (14)
C11A	0.0277 (18)	0.0311 (18)	0.0218 (16)	0.0030 (14)	0.0064 (14)	0.0042 (13)
C12A	0.042 (2)	0.0290 (18)	0.035 (2)	0.0035 (16)	0.0134 (17)	0.0081 (15)
C13A	0.0275 (18)	0.0289 (17)	0.0242 (17)	−0.0044 (14)	0.0067 (14)	−0.0029 (13)
C14A	0.0238 (18)	0.039 (2)	0.0276 (18)	0.0007 (15)	0.0073 (15)	0.0033 (15)
C15A	0.0201 (17)	0.052 (2)	0.0268 (18)	0.0008 (16)	0.0077 (15)	−0.0071 (16)
C16A	0.030 (2)	0.069 (3)	0.030 (2)	0.0015 (19)	0.0101 (17)	0.0015 (18)
C17A	0.034 (2)	0.088 (3)	0.028 (2)	0.000 (2)	0.0076 (18)	−0.021 (2)
C18A	0.043 (2)	0.063 (3)	0.046 (2)	0.001 (2)	0.011 (2)	−0.018 (2)
C19A	0.048 (2)	0.051 (2)	0.041 (2)	−0.002 (2)	0.0139 (19)	−0.0153 (18)
C20A	0.0280 (19)	0.043 (2)	0.033 (2)	−0.0020 (16)	0.0103 (16)	−0.0088 (16)
C21A	0.032 (2)	0.0351 (19)	0.032 (2)	−0.0004 (16)	0.0108 (16)	−0.0045 (16)
C22A	0.0253 (18)	0.0331 (18)	0.0270 (17)	0.0023 (14)	0.0065 (15)	0.0018 (14)
C23A	0.030 (2)	0.047 (2)	0.0321 (19)	−0.0074 (16)	0.0024 (16)	−0.0016 (16)
Br1B	0.0400 (2)	0.03334 (19)	0.0493 (2)	0.00394 (16)	0.01081 (18)	0.01429 (17)
Br2B	0.02973 (18)	0.03980 (19)	0.03393 (18)	−0.00315 (16)	0.00217 (14)	0.00055 (16)
O1B	0.0527 (17)	0.0392 (16)	0.0448 (16)	0.0032 (12)	0.0235 (14)	0.0156 (12)
O2B	0.0387 (15)	0.0312 (13)	0.0352 (14)	0.0064 (11)	0.0103 (12)	0.0097 (11)
N1B	0.0348 (16)	0.0324 (16)	0.0262 (15)	0.0013 (13)	0.0143 (13)	0.0062 (12)
C1B	0.0263 (17)	0.0190 (16)	0.0208 (15)	−0.0025 (12)	0.0040 (13)	−0.0025 (12)
C2B	0.0260 (16)	0.0220 (15)	0.0222 (15)	−0.0020 (13)	0.0069 (13)	−0.0037 (13)
C3B	0.0257 (17)	0.0216 (16)	0.0225 (16)	0.0028 (13)	0.0050 (14)	−0.0008 (12)
C4B	0.0316 (18)	0.0207 (16)	0.0233 (17)	0.0031 (13)	0.0078 (14)	0.0008 (13)
C5B	0.0286 (18)	0.0226 (16)	0.0237 (16)	−0.0018 (13)	0.0098 (14)	−0.0008 (13)
C6B	0.0250 (16)	0.0229 (15)	0.0249 (16)	−0.0033 (14)	0.0038 (13)	−0.0046 (13)
C7B	0.0294 (18)	0.0323 (18)	0.0255 (18)	−0.0010 (15)	0.0051 (15)	0.0039 (14)
C8B	0.0226 (17)	0.0338 (19)	0.0263 (17)	−0.0036 (14)	0.0055 (14)	0.0015 (13)
C9B	0.035 (2)	0.047 (2)	0.0301 (19)	−0.0016 (17)	0.0163 (17)	−0.0055 (16)
C10B	0.0308 (19)	0.0307 (18)	0.0270 (18)	0.0026 (14)	0.0053 (15)	0.0007 (14)
C11B	0.034 (2)	0.0302 (18)	0.0283 (18)	0.0065 (15)	0.0074 (15)	0.0040 (14)
C12B	0.060 (3)	0.026 (2)	0.036 (2)	0.003 (2)	0.014 (2)	0.0077 (18)
C11C	0.034 (2)	0.0302 (18)	0.0283 (18)	0.0065 (15)	0.0074 (15)	0.0040 (14)
C12C	0.060 (3)	0.026 (2)	0.036 (2)	0.003 (2)	0.014 (2)	0.0077 (18)
C13B	0.036 (2)	0.0332 (19)	0.0325 (19)	0.0000 (15)	0.0147 (16)	0.0054 (15)
C14B	0.031 (2)	0.040 (2)	0.034 (2)	0.0050 (16)	0.0170 (17)	0.0130 (16)
C15B	0.0236 (18)	0.050 (2)	0.0293 (19)	0.0047 (16)	0.0111 (15)	0.0062 (16)
C16B	0.034 (2)	0.059 (3)	0.033 (2)	0.0056 (18)	0.0145 (17)	0.0122 (19)
C17B	0.041 (2)	0.077 (3)	0.026 (2)	0.011 (2)	0.0108 (18)	0.004 (2)
C18B	0.039 (2)	0.066 (3)	0.034 (2)	0.005 (2)	0.0099 (18)	−0.0076 (19)
C19B	0.031 (2)	0.045 (2)	0.045 (2)	0.0006 (17)	0.0140 (18)	−0.0004 (17)
C20B	0.0223 (17)	0.040 (2)	0.0304 (19)	0.0032 (15)	0.0097 (15)	0.0057 (15)
C21B	0.0229 (17)	0.0359 (19)	0.0326 (19)	0.0040 (15)	0.0100 (15)	0.0045 (16)
C22B	0.0241 (17)	0.0311 (18)	0.0345 (19)	0.0001 (14)	0.0063 (15)	0.0018 (14)
C23B	0.029 (2)	0.063 (3)	0.057 (3)	−0.0114 (19)	0.0056 (19)	−0.004 (2)

### 2-[3,5-Bis(bromomethyl)-2,4,6-triethylbenzyl]isoindoline-1,3-dione (1) Geometric parameters (Å, º)

**Table:**

Br1A—C7A	1.983 (3)	N1B—C21B	1.392 (4)
Br2A—C10A	1.975 (3)	N1B—C14B	1.411 (4)
O1A—C14A	1.210 (4)	N1B—C13B	1.472 (4)
O2A—C21A	1.208 (4)	C1B—C2B	1.407 (4)
N1A—C21A	1.394 (4)	C1B—C6B	1.415 (4)
N1A—C14A	1.406 (4)	C1B—C7B	1.499 (4)
N1A—C13A	1.471 (4)	C2B—C3B	1.406 (4)
C1A—C2A	1.401 (4)	C2B—C8B	1.516 (4)
C1A—C6A	1.412 (4)	C3B—C4B	1.409 (4)
C1A—C7A	1.505 (4)	C3B—C10B	1.498 (4)
C2A—C3A	1.406 (4)	C4B—C5B	1.412 (4)
C2A—C8A	1.517 (4)	C4B—C11C	1.517 (4)
C3A—C4A	1.411 (4)	C4B—C11B	1.517 (4)
C3A—C10A	1.503 (4)	C5B—C6B	1.411 (4)
C4A—C5A	1.402 (4)	C5B—C13B	1.518 (4)
C4A—C11A	1.519 (4)	C6B—C22B	1.514 (4)
C5A—C6A	1.412 (4)	C7B—H7C	0.9900
C5A—C13A	1.528 (4)	C7B—H7D	0.9900
C6A—C22A	1.518 (4)	C8B—C9B	1.532 (4)
C7A—H7A	0.9900	C8B—H8C	0.9900
C7A—H7B	0.9900	C8B—H8D	0.9900
C8A—C9A	1.536 (5)	C9B—H9D	0.9800
C8A—H8A	0.9900	C9B—H9E	0.9800
C8A—H8B	0.9900	C9B—H9F	0.9800
C9A—H9A	0.9800	C10B—H10C	0.9900
C9A—H9B	0.9800	C10B—H10D	0.9900
C9A—H9C	0.9800	C11B—C12B	1.510 (5)
C10A—H10A	0.9900	C11B—H11C	0.9900
C10A—H10B	0.9900	C11B—H11D	0.9900
C11A—C12A	1.531 (5)	C12B—H12D	0.9800
C11A—H11A	0.9900	C12B—H12E	0.9800
C11A—H11B	0.9900	C12B—H12F	0.9800
C12A—H12A	0.9800	C11C—C12C	1.41 (2)
C12A—H12B	0.9800	C11C—H11E	0.9900
C12A—H12C	0.9800	C11C—H11F	0.9900
C13A—H13A	0.9900	C12C—H12G	0.9800
C13A—H13B	0.9900	C12C—H12H	0.9800
C14A—C15A	1.493 (5)	C12C—H12I	0.9800
C15A—C20A	1.380 (5)	C13B—H13C	0.9900
C15A—C16A	1.390 (5)	C13B—H13D	0.9900
C16A—C17A	1.414 (6)	C14B—C15B	1.496 (5)
C16A—H16A	0.9500	C15B—C16B	1.388 (5)
C17A—C18A	1.375 (6)	C15B—C20B	1.389 (5)
C17A—H17A	0.9500	C16B—C17B	1.392 (6)
C18A—C19A	1.384 (6)	C16B—H16B	0.9500
C18A—H18A	0.9500	C17B—C18B	1.380 (6)
C19A—C20A	1.387 (5)	C17B—H17B	0.9500
C19A—H19A	0.9500	C18B—C19B	1.398 (5)
C20A—C21A	1.491 (5)	C18B—H18B	0.9500
C22A—C23A	1.521 (5)	C19B—C20B	1.385 (5)
C22A—H22A	0.9900	C19B—H19B	0.9500
C22A—H22B	0.9900	C20B—C21B	1.488 (5)
C23A—H23A	0.9800	C22B—C23B	1.527 (5)
C23A—H23B	0.9800	C22B—H22C	0.9900
C23A—H23C	0.9800	C22B—H22D	0.9900
Br1B—C7B	1.979 (3)	C23B—H23D	0.9800
Br2B—C10B	1.980 (3)	C23B—H23E	0.9800
O1B—C14B	1.212 (4)	C23B—H23F	0.9800
O2B—C21B	1.213 (4)		
			
C21A—N1A—C14A	111.9 (3)	C3B—C2B—C8B	120.9 (3)
C21A—N1A—C13A	124.6 (3)	C1B—C2B—C8B	120.3 (3)
C14A—N1A—C13A	123.2 (3)	C2B—C3B—C4B	120.6 (3)
C2A—C1A—C6A	121.0 (3)	C2B—C3B—C10B	119.0 (3)
C2A—C1A—C7A	119.3 (3)	C4B—C3B—C10B	120.4 (3)
C6A—C1A—C7A	119.7 (3)	C3B—C4B—C5B	120.3 (3)
C1A—C2A—C3A	119.3 (3)	C3B—C4B—C11C	119.3 (3)
C1A—C2A—C8A	120.3 (3)	C5B—C4B—C11C	120.4 (3)
C3A—C2A—C8A	120.3 (3)	C3B—C4B—C11B	119.3 (3)
C2A—C3A—C4A	120.4 (3)	C5B—C4B—C11B	120.4 (3)
C2A—C3A—C10A	119.7 (3)	C6B—C5B—C4B	119.6 (3)
C4A—C3A—C10A	119.9 (3)	C6B—C5B—C13B	120.3 (3)
C5A—C4A—C3A	119.9 (3)	C4B—C5B—C13B	120.0 (3)
C5A—C4A—C11A	120.3 (3)	C5B—C6B—C1B	119.4 (3)
C3A—C4A—C11A	119.6 (3)	C5B—C6B—C22B	121.6 (3)
C4A—C5A—C6A	120.2 (3)	C1B—C6B—C22B	119.1 (3)
C4A—C5A—C13A	120.2 (3)	C1B—C7B—Br1B	112.3 (2)
C6A—C5A—C13A	119.5 (3)	C1B—C7B—H7C	109.1
C1A—C6A—C5A	119.1 (3)	Br1B—C7B—H7C	109.1
C1A—C6A—C22A	119.5 (3)	C1B—C7B—H7D	109.1
C5A—C6A—C22A	121.4 (3)	Br1B—C7B—H7D	109.1
C1A—C7A—Br1A	112.1 (2)	H7C—C7B—H7D	107.9
C1A—C7A—H7A	109.2	C2B—C8B—C9B	112.3 (3)
Br1A—C7A—H7A	109.2	C2B—C8B—H8C	109.1
C1A—C7A—H7B	109.2	C9B—C8B—H8C	109.1
Br1A—C7A—H7B	109.2	C2B—C8B—H8D	109.1
H7A—C7A—H7B	107.9	C9B—C8B—H8D	109.1
C2A—C8A—C9A	111.2 (3)	H8C—C8B—H8D	107.9
C2A—C8A—H8A	109.4	C8B—C9B—H9D	109.5
C9A—C8A—H8A	109.4	C8B—C9B—H9E	109.5
C2A—C8A—H8B	109.4	H9D—C9B—H9E	109.5
C9A—C8A—H8B	109.4	C8B—C9B—H9F	109.5
H8A—C8A—H8B	108.0	H9D—C9B—H9F	109.5
C8A—C9A—H9A	109.5	H9E—C9B—H9F	109.5
C8A—C9A—H9B	109.5	C3B—C10B—Br2B	110.8 (2)
H9A—C9A—H9B	109.5	C3B—C10B—H10C	109.5
C8A—C9A—H9C	109.5	Br2B—C10B—H10C	109.5
H9A—C9A—H9C	109.5	C3B—C10B—H10D	109.5
H9B—C9A—H9C	109.5	Br2B—C10B—H10D	109.5
C3A—C10A—Br2A	110.9 (2)	H10C—C10B—H10D	108.1
C3A—C10A—H10A	109.5	C12B—C11B—C4B	110.9 (3)
Br2A—C10A—H10A	109.5	C12B—C11B—H11C	109.5
C3A—C10A—H10B	109.5	C4B—C11B—H11C	109.5
Br2A—C10A—H10B	109.5	C12B—C11B—H11D	109.5
H10A—C10A—H10B	108.1	C4B—C11B—H11D	109.5
C4A—C11A—C12A	110.7 (3)	H11C—C11B—H11D	108.0
C4A—C11A—H11A	109.5	C11B—C12B—H12D	109.5
C12A—C11A—H11A	109.5	C11B—C12B—H12E	109.5
C4A—C11A—H11B	109.5	H12D—C12B—H12E	109.5
C12A—C11A—H11B	109.5	C11B—C12B—H12F	109.5
H11A—C11A—H11B	108.1	H12D—C12B—H12F	109.5
C11A—C12A—H12A	109.5	H12E—C12B—H12F	109.5
C11A—C12A—H12B	109.5	C12C—C11C—C4B	114.2 (8)
H12A—C12A—H12B	109.5	C12C—C11C—H11E	108.7
C11A—C12A—H12C	109.5	C4B—C11C—H11E	108.7
H12A—C12A—H12C	109.5	C12C—C11C—H11F	108.7
H12B—C12A—H12C	109.5	C4B—C11C—H11F	108.7
N1A—C13A—C5A	114.9 (3)	H11E—C11C—H11F	107.6
N1A—C13A—H13A	108.5	C11C—C12C—H12G	109.5
C5A—C13A—H13A	108.5	C11C—C12C—H12H	109.5
N1A—C13A—H13B	108.5	H12G—C12C—H12H	109.5
C5A—C13A—H13B	108.5	C11C—C12C—H12I	109.5
H13A—C13A—H13B	107.5	H12G—C12C—H12I	109.5
O1A—C14A—N1A	124.9 (3)	H12H—C12C—H12I	109.5
O1A—C14A—C15A	129.9 (3)	N1B—C13B—C5B	114.2 (3)
N1A—C14A—C15A	105.2 (3)	N1B—C13B—H13C	108.7
C20A—C15A—C16A	121.2 (3)	C5B—C13B—H13C	108.7
C20A—C15A—C14A	108.7 (3)	N1B—C13B—H13D	108.7
C16A—C15A—C14A	130.1 (4)	C5B—C13B—H13D	108.7
C15A—C16A—C17A	116.0 (4)	H13C—C13B—H13D	107.6
C15A—C16A—H16A	122.0	O1B—C14B—N1B	124.7 (3)
C17A—C16A—H16A	122.0	O1B—C14B—C15B	129.8 (3)
C18A—C17A—C16A	122.4 (4)	N1B—C14B—C15B	105.5 (3)
C18A—C17A—H17A	118.8	C16B—C15B—C20B	121.3 (4)
C16A—C17A—H17A	118.8	C16B—C15B—C14B	130.6 (3)
C17A—C18A—C19A	120.7 (4)	C20B—C15B—C14B	108.1 (3)
C17A—C18A—H18A	119.7	C15B—C16B—C17B	116.7 (4)
C19A—C18A—H18A	119.7	C15B—C16B—H16B	121.7
C18A—C19A—C20A	117.5 (4)	C17B—C16B—H16B	121.7
C18A—C19A—H19A	121.2	C18B—C17B—C16B	122.1 (4)
C20A—C19A—H19A	121.2	C18B—C17B—H17B	118.9
C15A—C20A—C19A	122.1 (3)	C16B—C17B—H17B	118.9
C15A—C20A—C21A	108.1 (3)	C17B—C18B—C19B	121.2 (4)
C19A—C20A—C21A	129.8 (4)	C17B—C18B—H18B	119.4
O2A—C21A—N1A	125.0 (3)	C19B—C18B—H18B	119.4
O2A—C21A—C20A	129.1 (3)	C20B—C19B—C18B	116.7 (4)
N1A—C21A—C20A	106.0 (3)	C20B—C19B—H19B	121.6
C6A—C22A—C23A	112.6 (3)	C18B—C19B—H19B	121.6
C6A—C22A—H22A	109.1	C19B—C20B—C15B	122.0 (3)
C23A—C22A—H22A	109.1	C19B—C20B—C21B	129.8 (3)
C6A—C22A—H22B	109.1	C15B—C20B—C21B	108.2 (3)
C23A—C22A—H22B	109.1	O2B—C21B—N1B	124.8 (3)
H22A—C22A—H22B	107.8	O2B—C21B—C20B	128.8 (3)
C22A—C23A—H23A	109.5	N1B—C21B—C20B	106.3 (3)
C22A—C23A—H23B	109.5	C6B—C22B—C23B	113.1 (3)
H23A—C23A—H23B	109.5	C6B—C22B—H22C	108.9
C22A—C23A—H23C	109.5	C23B—C22B—H22C	108.9
H23A—C23A—H23C	109.5	C6B—C22B—H22D	108.9
H23B—C23A—H23C	109.5	C23B—C22B—H22D	108.9
C21B—N1B—C14B	111.7 (3)	H22C—C22B—H22D	107.8
C21B—N1B—C13B	124.5 (3)	C22B—C23B—H23D	109.5
C14B—N1B—C13B	123.8 (3)	C22B—C23B—H23E	109.5
C2B—C1B—C6B	121.2 (3)	H23D—C23B—H23E	109.5
C2B—C1B—C7B	119.1 (3)	C22B—C23B—H23F	109.5
C6B—C1B—C7B	119.7 (3)	H23D—C23B—H23F	109.5
C3B—C2B—C1B	118.9 (3)	H23E—C23B—H23F	109.5
			
C6A—C1A—C2A—C3A	2.0 (5)	C7B—C1B—C2B—C8B	6.2 (4)
C7A—C1A—C2A—C3A	−175.9 (3)	C1B—C2B—C3B—C4B	−0.8 (4)
C6A—C1A—C2A—C8A	179.0 (3)	C8B—C2B—C3B—C4B	180.0 (3)
C7A—C1A—C2A—C8A	1.1 (4)	C1B—C2B—C3B—C10B	178.5 (3)
C1A—C2A—C3A—C4A	0.1 (5)	C8B—C2B—C3B—C10B	−0.7 (4)
C8A—C2A—C3A—C4A	−176.9 (3)	C2B—C3B—C4B—C5B	−1.9 (5)
C1A—C2A—C3A—C10A	179.0 (3)	C10B—C3B—C4B—C5B	178.8 (3)
C8A—C2A—C3A—C10A	2.0 (5)	C2B—C3B—C4B—C11C	175.8 (3)
C2A—C3A—C4A—C5A	−1.3 (4)	C10B—C3B—C4B—C11C	−3.5 (4)
C10A—C3A—C4A—C5A	179.8 (3)	C2B—C3B—C4B—C11B	175.8 (3)
C2A—C3A—C4A—C11A	174.2 (3)	C10B—C3B—C4B—C11B	−3.5 (4)
C10A—C3A—C4A—C11A	−4.7 (4)	C3B—C4B—C5B—C6B	1.3 (5)
C3A—C4A—C5A—C6A	0.4 (4)	C11C—C4B—C5B—C6B	−176.4 (3)
C11A—C4A—C5A—C6A	−175.1 (3)	C11B—C4B—C5B—C6B	−176.4 (3)
C3A—C4A—C5A—C13A	176.4 (3)	C3B—C4B—C5B—C13B	177.9 (3)
C11A—C4A—C5A—C13A	0.9 (4)	C11C—C4B—C5B—C13B	0.2 (4)
C2A—C1A—C6A—C5A	−2.9 (4)	C11B—C4B—C5B—C13B	0.2 (4)
C7A—C1A—C6A—C5A	175.0 (3)	C4B—C5B—C6B—C1B	1.9 (4)
C2A—C1A—C6A—C22A	178.5 (3)	C13B—C5B—C6B—C1B	−174.7 (3)
C7A—C1A—C6A—C22A	−3.6 (4)	C4B—C5B—C6B—C22B	−177.4 (3)
C4A—C5A—C6A—C1A	1.7 (4)	C13B—C5B—C6B—C22B	6.0 (4)
C13A—C5A—C6A—C1A	−174.3 (3)	C2B—C1B—C6B—C5B	−4.7 (4)
C4A—C5A—C6A—C22A	−179.8 (3)	C7B—C1B—C6B—C5B	172.4 (3)
C13A—C5A—C6A—C22A	4.2 (4)	C2B—C1B—C6B—C22B	174.7 (3)
C2A—C1A—C7A—Br1A	−89.3 (3)	C7B—C1B—C6B—C22B	−8.2 (4)
C6A—C1A—C7A—Br1A	92.8 (3)	C2B—C1B—C7B—Br1B	−90.8 (3)
C1A—C2A—C8A—C9A	−89.4 (4)	C6B—C1B—C7B—Br1B	92.0 (3)
C3A—C2A—C8A—C9A	87.6 (4)	C3B—C2B—C8B—C9B	86.8 (4)
C2A—C3A—C10A—Br2A	91.8 (3)	C1B—C2B—C8B—C9B	−92.4 (4)
C4A—C3A—C10A—Br2A	−89.3 (3)	C2B—C3B—C10B—Br2B	92.4 (3)
C5A—C4A—C11A—C12A	90.8 (3)	C4B—C3B—C10B—Br2B	−88.3 (3)
C3A—C4A—C11A—C12A	−84.7 (4)	C3B—C4B—C11B—C12B	−84.5 (4)
C21A—N1A—C13A—C5A	52.9 (4)	C5B—C4B—C11B—C12B	93.2 (4)
C14A—N1A—C13A—C5A	−133.3 (3)	C3B—C4B—C11C—C12C	87.7 (10)
C4A—C5A—C13A—N1A	73.6 (4)	C5B—C4B—C11C—C12C	−94.6 (10)
C6A—C5A—C13A—N1A	−110.4 (3)	C21B—N1B—C13B—C5B	54.5 (4)
C21A—N1A—C14A—O1A	177.5 (3)	C14B—N1B—C13B—C5B	−128.2 (3)
C13A—N1A—C14A—O1A	3.0 (5)	C6B—C5B—C13B—N1B	−113.3 (3)
C21A—N1A—C14A—C15A	−3.3 (4)	C4B—C5B—C13B—N1B	70.1 (4)
C13A—N1A—C14A—C15A	−177.8 (3)	C21B—N1B—C14B—O1B	178.8 (3)
O1A—C14A—C15A—C20A	−179.5 (4)	C13B—N1B—C14B—O1B	1.2 (5)
N1A—C14A—C15A—C20A	1.3 (4)	C21B—N1B—C14B—C15B	−2.2 (4)
O1A—C14A—C15A—C16A	2.2 (6)	C13B—N1B—C14B—C15B	−179.8 (3)
N1A—C14A—C15A—C16A	−176.9 (3)	O1B—C14B—C15B—C16B	0.6 (6)
C20A—C15A—C16A—C17A	−1.6 (5)	N1B—C14B—C15B—C16B	−178.4 (3)
C14A—C15A—C16A—C17A	176.5 (3)	O1B—C14B—C15B—C20B	−180.0 (4)
C15A—C16A—C17A—C18A	0.8 (6)	N1B—C14B—C15B—C20B	1.1 (4)
C16A—C17A—C18A—C19A	0.4 (6)	C20B—C15B—C16B—C17B	0.3 (5)
C17A—C18A—C19A—C20A	−0.8 (6)	C14B—C15B—C16B—C17B	179.7 (3)
C16A—C15A—C20A—C19A	1.3 (5)	C15B—C16B—C17B—C18B	−0.1 (6)
C14A—C15A—C20A—C19A	−177.2 (3)	C16B—C17B—C18B—C19B	0.0 (6)
C16A—C15A—C20A—C21A	179.4 (3)	C17B—C18B—C19B—C20B	0.0 (6)
C14A—C15A—C20A—C21A	0.9 (4)	C18B—C19B—C20B—C15B	0.2 (5)
C18A—C19A—C20A—C15A	0.0 (6)	C18B—C19B—C20B—C21B	180.0 (3)
C18A—C19A—C20A—C21A	−177.7 (4)	C16B—C15B—C20B—C19B	−0.4 (5)
C14A—N1A—C21A—O2A	−176.4 (3)	C14B—C15B—C20B—C19B	−179.9 (3)
C13A—N1A—C21A—O2A	−2.0 (6)	C16B—C15B—C20B—C21B	179.8 (3)
C14A—N1A—C21A—C20A	3.9 (4)	C14B—C15B—C20B—C21B	0.3 (4)
C13A—N1A—C21A—C20A	178.3 (3)	C14B—N1B—C21B—O2B	−177.6 (3)
C15A—C20A—C21A—O2A	177.4 (4)	C13B—N1B—C21B—O2B	0.0 (5)
C19A—C20A—C21A—O2A	−4.6 (7)	C14B—N1B—C21B—C20B	2.4 (4)
C15A—C20A—C21A—N1A	−2.9 (4)	C13B—N1B—C21B—C20B	179.9 (3)
C19A—C20A—C21A—N1A	175.0 (4)	C19B—C20B—C21B—O2B	−1.4 (6)
C1A—C6A—C22A—C23A	91.4 (4)	C15B—C20B—C21B—O2B	178.4 (3)
C5A—C6A—C22A—C23A	−87.1 (4)	C19B—C20B—C21B—N1B	178.7 (3)
C6B—C1B—C2B—C3B	4.1 (4)	C15B—C20B—C21B—N1B	−1.6 (4)
C7B—C1B—C2B—C3B	−173.0 (3)	C5B—C6B—C22B—C23B	−90.3 (4)
C6B—C1B—C2B—C8B	−176.7 (3)	C1B—C6B—C22B—C23B	90.3 (4)

### 2-[3,5-Bis(bromomethyl)-2,4,6-triethylbenzyl]isoindoline-1,3-dione (1) Hydrogen-bond geometry (Å, º)

**Table:**

*D*—H···*A*	*D*—H	H···*A*	*D*···*A*	*D*—H···*A*
C10*A*—H10*B*···O2*B*	0.99	2.35	3.223 (4)	147
C11*A*—H11*A*···N1*A*	0.99	2.54	3.283 (4)	132
C13*A*—H13*B*···Br2*B*^i^	0.99	2.92	3.746 (3)	142
C13*A*—H13*B*···O1*A*	0.99	2.52	2.914 (4)	103
C9*B*—H9*F*···Br2*A*^ii^	0.98	3.00	3.921 (4)	158
C11*B*—H11*D*···N1*B*	0.99	2.45	3.207 (4)	133
C12*B*—H12*D*···Br1*B*^ii^	0.98	2.86	3.499 (4)	123
C13*B*—H13*D*···O1*B*	0.99	2.53	2.928 (4)	104
C22*B*—H22*D*···O2*B*	0.99	2.64	3.322 (4)	126
C23*B*—H23*E*···O2*A*^iii^	0.98	2.43	3.226 (5)	138
C22*A*—H22*B*···O2*A*	0.99	2.59	3.278 (4)	126
C9*B*—H9*D*···*Cg*4^iv^	0.98	2.96	3.731 (5)	137
C23*B*—H23*D*···*Cg*4^v^	0.98	2.92	3.542 (5)	122
C12*C*—H12*I*···N1*B*	0.98	2.56	3.24 (2)	126

Symmetry codes: (i) *x*−1, *y*, *z*; (ii) −*x*+3/2, *y*−1/2, −*z*+1/2; (iii) −*x*+1/2, *y*−1/2, −*z*+1/2; (iv) *x*−1/2, −*y*+1/2, *z*−1/2; (v) *x*+1/2, −*y*+1/2, *z*−1/2.

### 2-{5-(Bromomethyl)-3-[(1,3-dioxoisoindolin-2-yl)methyl]-2,4,6-triethylbenzyl}isoindoline-1,3-dione (2) Crystal data

**Table:**

C_31_H_29_BrN_2_O_4_	*F*(000) = 1184
*M**_r_* = 573.47	*D*_x_ = 1.437 Mg m^−^^3^
Monoclinic, *P*2_1_/*n*	Mo *K*α radiation, λ = 0.71073 Å
*a* = 12.899 (2) Å	Cell parameters from 3744 reflections
*b* = 12.9748 (15) Å	θ = 2.0–22.5°
*c* = 16.763 (3) Å	µ = 1.59 mm^−^^1^
β = 109.168 (13)°	*T* = 153 K
*V* = 2649.9 (7) Å^3^	Piece, colorless
*Z* = 4	0.18 × 0.18 × 0.15 mm

### 2-{5-(Bromomethyl)-3-[(1,3-dioxoisoindolin-2-yl)methyl]-2,4,6-triethylbenzyl}isoindoline-1,3-dione (2) Data collection

**Table:**

STOE IPDS 2 diffractometer	4941 independent reflections
Radiation source: sealed X-ray tube, 12 x 0.4 mm long-fine focus	3442 reflections with *I* > 2σ(*I*)
Plane graphite monochromator	*R*_int_ = 0.115
Detector resolution: 6.67 pixels mm^-1^	θ_max_ = 25.5°, θ_min_ = 1.7°
rotation method scans	*h* = −15→15
Absorption correction: integration	*k* = −15→15
*T*_min_ = 0.695, *T*_max_ = 0.844	*l* = −19→20
26391 measured reflections	

### 2-{5-(Bromomethyl)-3-[(1,3-dioxoisoindolin-2-yl)methyl]-2,4,6-triethylbenzyl}isoindoline-1,3-dione (2) Refinement

**Table:**

Refinement on *F*^2^	0 restraints
Least-squares matrix: full	Hydrogen site location: inferred from neighbouring sites
*R*[*F*^2^ > 2σ(*F*^2^)] = 0.056	H-atom parameters constrained
*wR*(*F*^2^) = 0.129	*w* = 1/[σ^2^(*F*_o_^2^) + (0.0406*P*)^2^ + 4.7828*P*] where *P* = (*F*_o_^2^ + 2*F*_c_^2^)/3
*S* = 1.12	(Δ/σ)_max_ < 0.001
4941 reflections	Δρ_max_ = 0.38 e Å^−^^3^
346 parameters	Δρ_min_ = −0.67 e Å^−^^3^

### 2-{5-(Bromomethyl)-3-[(1,3-dioxoisoindolin-2-yl)methyl]-2,4,6-triethylbenzyl}isoindoline-1,3-dione (2) Special details

**Table:**

Geometry. All esds (except the esd in the dihedral angle between two l.s. planes) are estimated using the full covariance matrix. The cell esds are taken into account individually in the estimation of esds in distances, angles and torsion angles; correlations between esds in cell parameters are only used when they are defined by crystal symmetry. An approximate (isotropic) treatment of cell esds is used for estimating esds involving l.s. planes.

### 2-{5-(Bromomethyl)-3-[(1,3-dioxoisoindolin-2-yl)methyl]-2,4,6-triethylbenzyl}isoindoline-1,3-dione (2) Fractional atomic coordinates and isotropic or equivalent isotropic displacement parameters (Å^2^)

**Table:**

	*x*	*y*	*z*	*U*_iso_*/*U*_eq_	
Br1	0.52316 (4)	0.01014 (4)	0.79824 (3)	0.04165 (17)	
O1	0.0480 (2)	−0.1049 (2)	0.44957 (19)	0.0269 (7)	
O2	0.2500 (2)	0.1152 (2)	0.34544 (18)	0.0264 (7)	
O3	0.2841 (3)	0.5612 (2)	0.47753 (19)	0.0337 (7)	
O4	0.4148 (2)	0.4359 (2)	0.74727 (17)	0.0235 (6)	
N1	0.1509 (2)	0.0228 (2)	0.4147 (2)	0.0178 (7)	
N2	0.3553 (3)	0.4731 (2)	0.6045 (2)	0.0187 (7)	
C1	0.4494 (3)	0.1118 (3)	0.6341 (3)	0.0207 (8)	
C2	0.3495 (3)	0.0700 (3)	0.5826 (2)	0.0186 (8)	
C3	0.2686 (3)	0.1364 (3)	0.5330 (2)	0.0169 (8)	
C4	0.2886 (3)	0.2437 (3)	0.5314 (2)	0.0170 (8)	
C5	0.3903 (3)	0.2834 (3)	0.5827 (2)	0.0178 (8)	
C6	0.4692 (3)	0.2186 (3)	0.6364 (2)	0.0183 (8)	
C7	0.5375 (3)	0.0407 (3)	0.6872 (3)	0.0261 (10)	
H7A	0.610137	0.072469	0.695790	0.031*	
H7B	0.534643	−0.024818	0.656210	0.031*	
C8	0.3313 (3)	−0.0454 (3)	0.5824 (3)	0.0218 (9)	
H8A	0.251482	−0.059241	0.565489	0.026*	
H8B	0.365684	−0.072266	0.640352	0.026*	
C9	0.3788 (4)	−0.1030 (3)	0.5223 (3)	0.0303 (10)	
H9A	0.347178	−0.074907	0.465118	0.046*	
H9B	0.360849	−0.176407	0.521898	0.046*	
H9C	0.458681	−0.094380	0.541444	0.046*	
C10	0.1550 (3)	0.0942 (3)	0.4834 (2)	0.0205 (9)	
H10A	0.125136	0.058587	0.523338	0.025*	
H10B	0.106010	0.153176	0.459244	0.025*	
C11	0.0915 (3)	−0.0691 (3)	0.4011 (3)	0.0186 (8)	
C12	0.0940 (3)	−0.1109 (3)	0.3197 (2)	0.0189 (8)	
C13	0.0457 (3)	−0.2003 (3)	0.2775 (3)	0.0258 (10)	
H13	0.004436	−0.245548	0.300068	0.031*	
C14	0.0612 (4)	−0.2193 (3)	0.2012 (3)	0.0325 (11)	
H14	0.030282	−0.279843	0.170719	0.039*	
C15	0.1209 (4)	−0.1524 (3)	0.1672 (3)	0.0324 (11)	
H15	0.129331	−0.167964	0.114331	0.039*	
C16	0.1682 (3)	−0.0627 (3)	0.2106 (3)	0.0246 (9)	
H16	0.208922	−0.016774	0.188112	0.030*	
C17	0.1535 (3)	−0.0438 (3)	0.2871 (2)	0.0189 (8)	
C18	0.1925 (3)	0.0425 (3)	0.3490 (2)	0.0179 (8)	
C19	0.2003 (3)	0.3149 (3)	0.4773 (3)	0.0215 (9)	
H19A	0.161193	0.280095	0.423207	0.026*	
H19B	0.235232	0.377749	0.464336	0.026*	
C20	0.1163 (3)	0.3462 (3)	0.5208 (3)	0.0283 (10)	
H20A	0.062288	0.393705	0.484201	0.042*	
H20B	0.154562	0.380291	0.574660	0.042*	
H20C	0.078697	0.284601	0.531248	0.042*	
C21	0.4188 (3)	0.3966 (3)	0.5753 (3)	0.0202 (9)	
H21A	0.497534	0.406640	0.607674	0.024*	
H21B	0.409090	0.411132	0.515254	0.024*	
C22	0.2951 (3)	0.5522 (3)	0.5513 (3)	0.0246 (9)	
C23	0.2531 (3)	0.6184 (3)	0.6061 (3)	0.0219 (9)	
C24	0.1883 (4)	0.7063 (3)	0.5864 (3)	0.0308 (10)	
H24	0.160892	0.732223	0.530356	0.037*	
C25	0.1652 (4)	0.7548 (3)	0.6535 (3)	0.0335 (11)	
H25	0.119858	0.814329	0.642455	0.040*	
C26	0.2069 (4)	0.7180 (3)	0.7352 (3)	0.0373 (12)	
H26	0.191087	0.753832	0.779323	0.045*	
C27	0.2714 (4)	0.6298 (3)	0.7542 (3)	0.0284 (10)	
H27	0.299597	0.604064	0.810256	0.034*	
C28	0.2928 (3)	0.5812 (3)	0.6878 (3)	0.0227 (9)	
C29	0.3610 (3)	0.4885 (3)	0.6876 (2)	0.0194 (8)	
C30	0.5750 (3)	0.2621 (3)	0.6972 (3)	0.0208 (9)	
H30A	0.601244	0.216141	0.746913	0.025*	
H30B	0.559601	0.330376	0.717248	0.025*	
C31	0.6659 (3)	0.2740 (3)	0.6580 (3)	0.0257 (9)	
H31A	0.686331	0.205933	0.642623	0.039*	
H31B	0.730070	0.306456	0.698949	0.039*	
H31C	0.639585	0.317153	0.607382	0.039*	

### 2-{5-(Bromomethyl)-3-[(1,3-dioxoisoindolin-2-yl)methyl]-2,4,6-triethylbenzyl}isoindoline-1,3-dione (2) Atomic displacement parameters (Å^2^)

**Table:**

	*U*^11^	*U*^22^	*U*^33^	*U*^12^	*U*^13^	*U*^23^
Br1	0.0519 (3)	0.0366 (3)	0.0270 (2)	0.0005 (2)	0.0001 (2)	0.0070 (2)
O1	0.0267 (15)	0.0224 (15)	0.0341 (17)	−0.0046 (13)	0.0132 (14)	−0.0026 (13)
O2	0.0301 (16)	0.0216 (15)	0.0251 (16)	−0.0051 (13)	0.0057 (13)	0.0031 (12)
O3	0.054 (2)	0.0249 (16)	0.0203 (17)	0.0032 (15)	0.0104 (15)	0.0044 (12)
O4	0.0284 (15)	0.0184 (14)	0.0218 (15)	0.0000 (12)	0.0054 (13)	0.0021 (12)
N1	0.0131 (15)	0.0174 (16)	0.0216 (17)	−0.0032 (13)	0.0040 (13)	−0.0006 (13)
N2	0.0244 (17)	0.0112 (15)	0.0202 (17)	0.0039 (13)	0.0070 (14)	0.0002 (13)
C1	0.021 (2)	0.0183 (19)	0.022 (2)	0.0021 (16)	0.0072 (17)	−0.0005 (16)
C2	0.0200 (19)	0.0159 (19)	0.021 (2)	−0.0025 (16)	0.0078 (17)	−0.0030 (15)
C3	0.0160 (19)	0.0176 (19)	0.0160 (19)	−0.0026 (15)	0.0038 (16)	−0.0032 (15)
C4	0.0193 (19)	0.0186 (19)	0.0132 (19)	0.0020 (16)	0.0054 (15)	−0.0022 (15)
C5	0.021 (2)	0.0120 (18)	0.021 (2)	0.0005 (15)	0.0064 (17)	−0.0021 (15)
C6	0.0181 (19)	0.0155 (18)	0.021 (2)	−0.0039 (15)	0.0059 (16)	−0.0028 (16)
C7	0.026 (2)	0.020 (2)	0.026 (2)	0.0007 (17)	0.0008 (18)	−0.0013 (17)
C8	0.024 (2)	0.0161 (18)	0.022 (2)	−0.0031 (16)	0.0028 (18)	0.0011 (15)
C9	0.038 (3)	0.018 (2)	0.030 (2)	0.0044 (19)	0.005 (2)	−0.0017 (18)
C10	0.0176 (19)	0.020 (2)	0.021 (2)	0.0004 (16)	0.0023 (17)	−0.0056 (16)
C11	0.0125 (18)	0.0164 (19)	0.024 (2)	0.0012 (15)	0.0021 (17)	0.0038 (16)
C12	0.0157 (19)	0.0167 (19)	0.023 (2)	0.0033 (16)	0.0042 (16)	−0.0019 (16)
C13	0.022 (2)	0.0162 (19)	0.035 (3)	−0.0004 (16)	0.0039 (19)	−0.0028 (17)
C14	0.032 (2)	0.025 (2)	0.033 (3)	0.0010 (19)	0.000 (2)	−0.0104 (19)
C15	0.040 (3)	0.031 (2)	0.022 (2)	0.007 (2)	0.004 (2)	−0.0060 (19)
C16	0.024 (2)	0.027 (2)	0.019 (2)	0.0048 (17)	0.0015 (17)	0.0058 (17)
C17	0.0156 (19)	0.0180 (19)	0.019 (2)	0.0040 (15)	−0.0001 (16)	0.0032 (15)
C18	0.0160 (19)	0.0159 (19)	0.018 (2)	0.0032 (15)	−0.0002 (16)	0.0034 (15)
C19	0.022 (2)	0.0172 (19)	0.023 (2)	−0.0003 (16)	0.0041 (17)	0.0011 (16)
C20	0.023 (2)	0.022 (2)	0.038 (3)	0.0052 (18)	0.007 (2)	0.0009 (19)
C21	0.025 (2)	0.0137 (18)	0.021 (2)	−0.0018 (16)	0.0063 (17)	−0.0041 (15)
C22	0.026 (2)	0.0132 (19)	0.033 (3)	−0.0029 (17)	0.0073 (19)	−0.0015 (17)
C23	0.022 (2)	0.0153 (19)	0.027 (2)	−0.0029 (16)	0.0066 (18)	−0.0007 (16)
C24	0.033 (2)	0.019 (2)	0.040 (3)	0.0007 (18)	0.012 (2)	0.0022 (19)
C25	0.030 (2)	0.017 (2)	0.057 (3)	0.0038 (18)	0.020 (2)	−0.003 (2)
C26	0.038 (3)	0.030 (2)	0.053 (3)	0.001 (2)	0.027 (2)	−0.008 (2)
C27	0.033 (2)	0.027 (2)	0.031 (2)	−0.0021 (19)	0.017 (2)	−0.0028 (19)
C28	0.024 (2)	0.0156 (18)	0.030 (2)	−0.0036 (16)	0.0101 (18)	−0.0024 (17)
C29	0.0222 (19)	0.0147 (18)	0.022 (2)	−0.0061 (16)	0.0087 (16)	−0.0017 (17)
C30	0.019 (2)	0.0180 (19)	0.021 (2)	−0.0012 (16)	0.0007 (17)	−0.0048 (16)
C31	0.024 (2)	0.019 (2)	0.033 (2)	0.0019 (17)	0.0073 (19)	−0.0040 (17)

### 2-{5-(Bromomethyl)-3-[(1,3-dioxoisoindolin-2-yl)methyl]-2,4,6-triethylbenzyl}isoindoline-1,3-dione (2) Geometric parameters (Å, º)

**Table:**

Br1—C7	1.970 (4)	C13—C14	1.381 (6)
O1—C11	1.221 (5)	C13—H13	0.9500
O2—C18	1.213 (5)	C14—C15	1.399 (7)
O3—C22	1.204 (5)	C14—H14	0.9500
O4—C29	1.222 (5)	C15—C16	1.401 (6)
N1—C11	1.395 (5)	C15—H15	0.9500
N1—C18	1.399 (5)	C16—C17	1.380 (6)
N1—C10	1.465 (5)	C16—H16	0.9500
N2—C29	1.384 (5)	C17—C18	1.497 (5)
N2—C22	1.413 (5)	C19—C20	1.547 (6)
N2—C21	1.471 (5)	C19—H19A	0.9900
C1—C2	1.403 (5)	C19—H19B	0.9900
C1—C6	1.407 (5)	C20—H20A	0.9800
C1—C7	1.507 (5)	C20—H20B	0.9800
C2—C3	1.397 (5)	C20—H20C	0.9800
C2—C8	1.515 (5)	C21—H21A	0.9900
C3—C4	1.418 (5)	C21—H21B	0.9900
C3—C10	1.529 (5)	C22—C23	1.483 (6)
C4—C5	1.409 (5)	C23—C28	1.383 (6)
C4—C19	1.515 (5)	C23—C24	1.388 (6)
C5—C6	1.396 (5)	C24—C25	1.403 (7)
C5—C21	1.530 (5)	C24—H24	0.9500
C6—C30	1.519 (5)	C25—C26	1.382 (7)
C7—H7A	0.9900	C25—H25	0.9500
C7—H7B	0.9900	C26—C27	1.389 (6)
C8—C9	1.534 (6)	C26—H26	0.9500
C8—H8A	0.9900	C27—C28	1.383 (6)
C8—H8B	0.9900	C27—H27	0.9500
C9—H9A	0.9800	C28—C29	1.491 (5)
C9—H9B	0.9800	C30—C31	1.528 (6)
C9—H9C	0.9800	C30—H30A	0.9900
C10—H10A	0.9900	C30—H30B	0.9900
C10—H10B	0.9900	C31—H31A	0.9800
C11—C12	1.478 (6)	C31—H31B	0.9800
C12—C17	1.386 (6)	C31—H31C	0.9800
C12—C13	1.394 (5)		
			
C11—N1—C18	111.1 (3)	C17—C16—C15	117.4 (4)
C11—N1—C10	123.1 (3)	C17—C16—H16	121.3
C18—N1—C10	125.4 (3)	C15—C16—H16	121.3
C29—N2—C22	111.5 (3)	C16—C17—C12	121.2 (4)
C29—N2—C21	125.6 (3)	C16—C17—C18	131.3 (4)
C22—N2—C21	122.0 (3)	C12—C17—C18	107.5 (3)
C2—C1—C6	121.3 (3)	O2—C18—N1	125.4 (4)
C2—C1—C7	119.3 (3)	O2—C18—C17	128.3 (4)
C6—C1—C7	119.5 (3)	N1—C18—C17	106.3 (3)
C3—C2—C1	118.9 (3)	C4—C19—C20	112.6 (3)
C3—C2—C8	121.5 (3)	C4—C19—H19A	109.1
C1—C2—C8	119.6 (4)	C20—C19—H19A	109.1
C2—C3—C4	120.8 (3)	C4—C19—H19B	109.1
C2—C3—C10	119.9 (3)	C20—C19—H19B	109.1
C4—C3—C10	119.3 (3)	H19A—C19—H19B	107.8
C5—C4—C3	119.1 (3)	C19—C20—H20A	109.5
C5—C4—C19	120.6 (3)	C19—C20—H20B	109.5
C3—C4—C19	120.3 (3)	H20A—C20—H20B	109.5
C6—C5—C4	120.6 (3)	C19—C20—H20C	109.5
C6—C5—C21	119.1 (3)	H20A—C20—H20C	109.5
C4—C5—C21	120.1 (3)	H20B—C20—H20C	109.5
C5—C6—C1	119.2 (3)	N2—C21—C5	116.5 (3)
C5—C6—C30	120.8 (3)	N2—C21—H21A	108.2
C1—C6—C30	120.0 (3)	C5—C21—H21A	108.2
C1—C7—Br1	113.3 (3)	N2—C21—H21B	108.2
C1—C7—H7A	108.9	C5—C21—H21B	108.2
Br1—C7—H7A	108.9	H21A—C21—H21B	107.3
C1—C7—H7B	108.9	O3—C22—N2	125.0 (4)
Br1—C7—H7B	108.9	O3—C22—C23	129.5 (4)
H7A—C7—H7B	107.7	N2—C22—C23	105.5 (4)
C2—C8—C9	112.8 (3)	C28—C23—C24	121.3 (4)
C2—C8—H8A	109.0	C28—C23—C22	108.7 (3)
C9—C8—H8A	109.0	C24—C23—C22	130.0 (4)
C2—C8—H8B	109.0	C23—C24—C25	116.6 (4)
C9—C8—H8B	109.0	C23—C24—H24	121.7
H8A—C8—H8B	107.8	C25—C24—H24	121.7
C8—C9—H9A	109.5	C26—C25—C24	121.6 (4)
C8—C9—H9B	109.5	C26—C25—H25	119.2
H9A—C9—H9B	109.5	C24—C25—H25	119.2
C8—C9—H9C	109.5	C25—C26—C27	121.3 (4)
H9A—C9—H9C	109.5	C25—C26—H26	119.3
H9B—C9—H9C	109.5	C27—C26—H26	119.3
N1—C10—C3	115.7 (3)	C28—C27—C26	117.0 (4)
N1—C10—H10A	108.3	C28—C27—H27	121.5
C3—C10—H10A	108.3	C26—C27—H27	121.5
N1—C10—H10B	108.3	C23—C28—C27	122.1 (4)
C3—C10—H10B	108.3	C23—C28—C29	107.8 (4)
H10A—C10—H10B	107.4	C27—C28—C29	130.1 (4)
O1—C11—N1	124.3 (4)	O4—C29—N2	124.9 (4)
O1—C11—C12	129.0 (3)	O4—C29—C28	128.7 (4)
N1—C11—C12	106.7 (3)	N2—C29—C28	106.4 (3)
C17—C12—C13	122.4 (4)	C6—C30—C31	113.3 (3)
C17—C12—C11	108.4 (3)	C6—C30—H30A	108.9
C13—C12—C11	129.2 (4)	C31—C30—H30A	108.9
C14—C13—C12	116.2 (4)	C6—C30—H30B	108.9
C14—C13—H13	121.9	C31—C30—H30B	108.9
C12—C13—H13	121.9	H30A—C30—H30B	107.7
C13—C14—C15	122.2 (4)	C30—C31—H31A	109.5
C13—C14—H14	118.9	C30—C31—H31B	109.5
C15—C14—H14	118.9	H31A—C31—H31B	109.5
C14—C15—C16	120.6 (4)	C30—C31—H31C	109.5
C14—C15—H15	119.7	H31A—C31—H31C	109.5
C16—C15—H15	119.7	H31B—C31—H31C	109.5
			
C6—C1—C2—C3	−0.1 (6)	C13—C12—C17—C16	−0.3 (6)
C7—C1—C2—C3	−179.5 (4)	C11—C12—C17—C16	−179.1 (3)
C6—C1—C2—C8	−179.6 (4)	C13—C12—C17—C18	179.9 (3)
C7—C1—C2—C8	1.0 (6)	C11—C12—C17—C18	1.1 (4)
C1—C2—C3—C4	3.0 (6)	C11—N1—C18—O2	−177.6 (3)
C8—C2—C3—C4	−177.5 (4)	C10—N1—C18—O2	10.3 (6)
C1—C2—C3—C10	−173.8 (4)	C11—N1—C18—C17	1.3 (4)
C8—C2—C3—C10	5.8 (6)	C10—N1—C18—C17	−170.8 (3)
C2—C3—C4—C5	−2.1 (6)	C16—C17—C18—O2	−2.3 (7)
C10—C3—C4—C5	174.7 (4)	C12—C17—C18—O2	177.4 (4)
C2—C3—C4—C19	179.9 (4)	C16—C17—C18—N1	178.8 (4)
C10—C3—C4—C19	−3.3 (6)	C12—C17—C18—N1	−1.5 (4)
C3—C4—C5—C6	−1.9 (6)	C5—C4—C19—C20	−95.5 (4)
C19—C4—C5—C6	176.1 (4)	C3—C4—C19—C20	82.5 (4)
C3—C4—C5—C21	173.4 (3)	C29—N2—C21—C5	68.4 (5)
C19—C4—C5—C21	−8.6 (6)	C22—N2—C21—C5	−123.2 (4)
C4—C5—C6—C1	4.7 (6)	C6—C5—C21—N2	−116.0 (4)
C21—C5—C6—C1	−170.6 (4)	C4—C5—C21—N2	68.6 (5)
C4—C5—C6—C30	−174.8 (4)	C29—N2—C22—O3	174.7 (4)
C21—C5—C6—C30	9.8 (6)	C21—N2—C22—O3	4.8 (6)
C2—C1—C6—C5	−3.8 (6)	C29—N2—C22—C23	−4.4 (4)
C7—C1—C6—C5	175.6 (4)	C21—N2—C22—C23	−174.2 (3)
C2—C1—C6—C30	175.8 (4)	O3—C22—C23—C28	−176.0 (4)
C7—C1—C6—C30	−4.8 (6)	N2—C22—C23—C28	2.9 (4)
C2—C1—C7—Br1	−87.0 (4)	O3—C22—C23—C24	1.7 (7)
C6—C1—C7—Br1	93.6 (4)	N2—C22—C23—C24	−179.3 (4)
C3—C2—C8—C9	96.5 (5)	C28—C23—C24—C25	−0.1 (6)
C1—C2—C8—C9	−84.0 (5)	C22—C23—C24—C25	−177.6 (4)
C11—N1—C10—C3	135.1 (4)	C23—C24—C25—C26	1.3 (6)
C18—N1—C10—C3	−53.7 (5)	C24—C25—C26—C27	−1.6 (7)
C2—C3—C10—N1	−66.4 (5)	C25—C26—C27—C28	0.6 (7)
C4—C3—C10—N1	116.7 (4)	C24—C23—C28—C27	−0.9 (6)
C18—N1—C11—O1	178.9 (3)	C22—C23—C28—C27	177.1 (4)
C10—N1—C11—O1	−8.8 (5)	C24—C23—C28—C29	−178.6 (4)
C18—N1—C11—C12	−0.7 (4)	C22—C23—C28—C29	−0.6 (4)
C10—N1—C11—C12	171.6 (3)	C26—C27—C28—C23	0.6 (6)
O1—C11—C12—C17	−179.8 (4)	C26—C27—C28—C29	177.8 (4)
N1—C11—C12—C17	−0.3 (4)	C22—N2—C29—O4	−174.3 (4)
O1—C11—C12—C13	1.4 (7)	C21—N2—C29—O4	−4.9 (6)
N1—C11—C12—C13	−179.1 (4)	C22—N2—C29—C28	4.0 (4)
C17—C12—C13—C14	0.6 (6)	C21—N2—C29—C28	173.5 (3)
C11—C12—C13—C14	179.3 (4)	C23—C28—C29—O4	176.3 (4)
C12—C13—C14—C15	−0.7 (6)	C27—C28—C29—O4	−1.3 (7)
C13—C14—C15—C16	0.4 (7)	C23—C28—C29—N2	−2.0 (4)
C14—C15—C16—C17	0.0 (6)	C27—C28—C29—N2	−179.5 (4)
C15—C16—C17—C12	−0.1 (6)	C5—C6—C30—C31	−87.0 (5)
C15—C16—C17—C18	179.7 (4)	C1—C6—C30—C31	93.4 (5)

### 2-{5-(Bromomethyl)-3-[(1,3-dioxoisoindolin-2-yl)methyl]-2,4,6-triethylbenzyl}isoindoline-1,3-dione (2) Hydrogen-bond geometry (Å, º)

*Cg*1 and *Cg*3 are the centroids of the C1–C6 and C12–C17 rings, respectively.

**Table:**

*D*—H···*A*	*D*—H	H···*A*	*D*···*A*	*D*—H···*A*
C10—H10*A*···O1	0.99	2.49	2.896 (5)	104
C10—H10*A*···O1^i^	0.99	2.49	3.173 (5)	126
C19—H19*B*···O3	0.99	2.45	3.373 (5)	154
C21—H21*B*···O3	0.99	2.47	2.897 (5)	105
C25—H25···O4^ii^	0.95	2.58	3.237 (5)	127
C30—H30*B*···O4	0.99	2.50	3.346 (5)	144
C31—H31*B*···O2^iii^	0.98	2.59	3.298 (5)	129
C31—H31*C*···O3^iv^	0.98	2.53	3.334 (5)	139
C26—H26···*Cg*1^ii^	0.95	2.84	3.529 (5)	130
C31—H31*A*···*Cg*3^v^	0.98	2.88	3.394 (5)	113

Symmetry codes: (i) −*x*, −*y*, −*z*+1; (ii) −*x*+1/2, *y*+1/2, −*z*+3/2; (iii) *x*+1/2, −*y*+1/2, *z*+1/2; (iv) −*x*+1, −*y*+1, −*z*+1; (v) −*x*+1, −*y*, −*z*+1.

## References

[bb1] Amrhein, F., Lippe, J. & Mazik, M. (2016). *Org. Biomol. Chem.* **14**, 10648–10659. 10.1039/c6ob01682k 27782281

[bb28] Amrhein, F. & Mazik, M. (2021). *Eur. J. Org. Chem.* https://chemistry-europe.onlinelibrary. wiley. com/doi/10.1002/ejoc.202100758.

[bb2] Bernstein, J., Davis, R. E., Shimoni, L. & Chang, N.-L. (1995). *Angew. Chem. Int. Ed. Engl.* **34**, 1555–1573.

[bb3] Bondi, A. (1964). *J. Phys. Chem.* **68**, 441–451.

[bb4] Chin, J., Oh, J., Jon, S. Y., Park, S. H., Walsdorff, C., Stranix, B., Ghoussoub, A., Lee, S. J., Chung, H. J., Park, S.-M. & Kim, K. (2002). *J. Am. Chem. Soc.* **124**, 5374–5379. 10.1021/ja0174175 11996578

[bb5] Domínguez, Z., Jancik, V., Leyva, M. A., Salas-Reyes, M., Guzmán-Márquez, V., Hernández, J., Bagatella-Flores, N. & Ramos, R. (2007). *Z. Kristallogr. New Cryst. Struct.* **222**, 146–148.

[bb6] Etter, M. C. (1990). *Acc. Chem. Res.* **23**, 120–126.

[bb7] Farrugia, L. J. (2012). *J. Appl. Cryst.* **45**, 849–854.

[bb8] Groom, C. R., Bruno, I. J., Lightfoot, M. P. & Ward, S. C. (2016). *Acta Cryst.* B**72**, 171–179. 10.1107/S2052520616003954 PMC4822653 27048719

[bb9] Hübschle, C. B., Sheldrick, G. M. & Dittrich, B. (2011). *J. Appl. Cryst.* **44**, 1281–1284. 10.1107/S0021889811043202 PMC3246833 22477785

[bb10] Jonah, T. M., Mathivathanan, L., Morozov, A. N., Mebel, A. M., Raptis, R. G. & Kavallieratos, K. (2017). *New J. Chem.* **41**, 14835–14838.

[bb11] Kaiser, S., Geffert, C. & Mazik, M. (2019). *Eur. J. Org. Chem.* pp. 7555–7562.

[bb12] Koch, N., Seichter, W. & Mazik, M. (2014). *Acta Cryst.* E**70**, o393–o394. 10.1107/S1600536814004383 PMC3998546 24826115

[bb29] Koch, N., Seichter, W. & Mazik, M. (2017). *CrystEngComm*, **19**, 3817–3833.

[bb13] Köhler, L., Seichter, W. & Mazik, M. (2020). *Eur. J. Org. Chem.* pp. 7023–7034.

[bb14] Lippe, J. & Mazik, M. (2013). *J. Org. Chem.* **78**, 9013–9020. 10.1021/jo400933q 24000949

[bb15] Lippe, J. & Mazik, M. (2015). *J. Org. Chem.* **80**, 1427–1439. 10.1021/jo502335u 25531805

[bb16] Mazik, M. (2009). *Chem. Soc. Rev.* **38**, 935–956. 10.1039/b710910p 19421573

[bb17] Mazik, M. (2012). *RSC Adv.* **2**, 2630–2642.

[bb18] Rosien, J.-R., Seichter, W. & Mazik, M. (2013). *Acta Cryst.* E**69**, o680. 10.1107/S1600536813008428 PMC3647874 23723840

[bb19] Schulze, M., Koch, N., Seichter, W. & Mazik, M. (2018). *Eur. J. Org. Chem.* pp. 4317–4330.

[bb30] Schulze, M., Schwarzer, A. & Mazik, M. (2017). *CrystEngComm*, **19**, 4003–4016.

[bb20] Seidel, P., Seichter, W., Schwarzer, A. & Mazik, M. (2021). *Eur. J. Org. Chem.* pp. 2901–2914.

[bb21] Sheldrick, G. M. (2008). *Acta Cryst.* A**64**, 112–122. 10.1107/S0108767307043930 18156677

[bb22] Sheldrick, G. M. (2015). *Acta Cryst.* C**71**, 3–8.

[bb23] Stapf, M., Seichter, W. & Mazik, M. (2015). *Chem. Eur. J.* **21**, 6350–6354. 10.1002/chem.201406383 25756753

[bb24] Stapf, M., Seichter, W. & Mazik, M. (2020*a*). *Eur. J. Org. Chem.* pp. 4900–4915.

[bb25] Stapf, M., Seichter, W. & Mazik, M. (2020*b*). *Acta Cryst.* E**76**, 1679–1683. 10.1107/S2056989020012554 PMC7534252 33117589

[bb26] Stoe (2009). *X-RED* and *X-AREA*. Stoe & Cie, Darmstadt, Germany.

[bb27] Westrip, S. P. (2010). *J. Appl. Cryst.* **43**, 920–925.

